# Experimental dissolution of road dust in simulated environmental and biological fluids

**DOI:** 10.1007/s11356-026-37727-7

**Published:** 2026-05-07

**Authors:** Ahmad Kamal Mubarok, Reto Gieré

**Affiliations:** 1 Department of Earth and Environmental Science, University of Pennsylvania, Philadelphia, PA USA; 2 Center of Excellence of Environmental Toxicology, University of Pennsylvania, Philadelphia, PA USA

**Keywords:** Road dust, Time-dependent leaching, EPA 3050B, Synthetic rainwater, Simulated gastric fluid, Gamble’s solution, Heavy metals, Bioaccessibility

## Abstract

Road dust from six sites in Philadelphia (PA, USA) was subjected to time-series dissolution experiments using the EPA 3050B method, synthetic rainwater, simulated gastric fluid, and Gamble's solution (simulated lung fluid) in order to study environmental availability and bioaccessibility of selected metals (Al, Fe, V, Cr, Ni, Cu, Zn, Pb). In general, the results showed that increasing acidity led to enhanced metal release, and that larger amounts of a given element were leached from the fine (< 75 µm) than the coarse (< 841 µm) size fraction of the same sample. Moreover, the higher the initial bulk metal content in the road dust, the higher the amount extracted. In simulated gastric fluid, many elements showed classic logarithmic concentration *vs.* time trends. In synthetic rainwater, only a few elements at some sites displayed such a pattern; rather, several metals exhibited a maximum concentration before the endpoint of the experiments, which is probably due to re-precipitation. In Gamble's solution, some samples released Al, V, Cu, and Zn, whereby distinct leaching behaviors of Al and V point to the presence of minor Al- and V-phases at some of the sites. The wide variability in concentration–time trends of the released metals as well as in their *potential* environmental availability, their environmental availability, and their bioaccessibility is most likely due to mineralogical differences between the sites. These differences document that it is problematic to draw general conclusions about possible environmental and health impacts of road dust, unless the speciation of potentially toxic elements is known. Our study further suggests that the maximum element-extraction rates may help in evaluating the effects of speciation on leaching behavior and duration as well as on potentially associated health risks subsequent to unintentional ingestion or inhalation of road-dust particles.

**Supplementary Information:**

The online version contains supplementary material available at 10.1007/s11356-026-37727-7.

## Introduction

Road dust originates from a range of activities and sources, including industrial processes, traffic (exhaust emissions; non-exhaust emissions due to abrasion of road surface, brakes and tires), soils, and deposition of atmospheric pollutants (Thorpe and Harrison 2008; Dietrich et al. 2022). As road dust particularly accumulates in densely populated urban areas, where people are exposed to it on a daily basis, it has the potential to pose significant health hazards because it can contain elevated levels of heavy metals as well as toxic organic components (Dietrich et al. 2022). The road-dust particles are dispersed and mobilized through traffic, wind, and stormwater into the surrounding environments and thus, become sinks or secondary sources of contaminants (Dietrich et al. 2022). Most geochemical studies of urban road dust have focused on its heavy metal and metalloid contents across different spatial and geological conditions (*e.g.,* Tanner et al. 2008; Wei et al. 2015; Dietrich et al. 2018; O'Shea et al. 2020; Wiseman et al. 2021; Zuo et al. 2022; Mostafa et al. 2024). More recently, however, the focus of research on urban road dust has shifted towards other contaminants, namely tire-abrasion particles, which are classified as microplastics, 6PPD-quinone from tire wear, and polycyclic aromatic hydrocarbons (*e.g.,* Sommer et al. 2018; Gieré and Dietze 2022; Rybak et al. 2022; Morioka et al. 2023; Gieré et al. 2024; Zhang et al. 2024; Edwards et al. 2025; Mohamed et al. 2025; Wang et al. 2025). Despite the growing interest in tire-related pollutants, investigations on both content and behavior of heavy metals in road dust remain relevant, as they help in assessing their potential impacts on environmental quality and human health.

Here, we study road dust from Philadelphia, which has been shown to contain high concentrations of heavy metals, with a mean of 119 ppm of total chromium (Cr), 355 ppm of cobalt (Co), and 516 ppm of lead (Pb), primarily derived from anthropogenic sources (O'Shea et al. 2020). These concentrations are substantially higher compared to other cities in the world, such as Beijing (China), with 84.7 ppm of Cr and 105 ppm of Pb (Wei et al. 2015); Hong Kong (China), with 324 ppm of Cr, 10.2 ppm of Co, and 240 ppm of Pb (Tanner et al. 2008); Middletown (Ohio, USA), with 160 ppm of Cr, 8.7 ppm of Co, and 85 ppm of Pb (Dietrich et al. 2018); and Cairo (Egypt), with 26 ppm of Cr, 15 ppm of Co, and 66 ppm of Pb (Mostafa et al. 2024). These high levels of heavy metals in Philadelphia, the sixth-largest city in the United States, probably resulted from the city’s history of extensive industrial activity, including paint and textile production, chemical processing, locomotive- and ship-building, and metalworking (Scranton 1989), from which these elements were likely released to local environmental media, such as road dust and soils (*e.g.*, Lusby et al. 2015; O'Shea et al. 2020). Additionally, the road dust in Philadelphia also contains natural materials (*e.g.*, various minerals) derived from the local geological formations (O’Shea et al. 2020).

Because humans can be exposed to road dust unintentionally through outer body contact, inhalation, and ingestion (*e.g.,* Tan et al. 2018), and because this material may contain high levels of contaminants, it is important to explore the potential impacts of road dust on human health (Zuo et al. 2022; Mostafa et al. 2024). Understanding how these exposures affect the human body could benefit human health assessment, and there is a growing body of literature devoted to this topic. Kastury et al. (2017), for example, reported that finer particles are generally more harmful as they travel farther into the respiratory tract and become more challenging to remove from the body, whereas Shi et al. (2011) suggested that, in general, ingestion represented the highest risk associated with road-dust exposure. Other researchers have investigated element distributions between road dust and simulated biological fluids to assess bioaccessibility of some components hosted by the bulk road dust or by its inhalable or ingestible particle-size fractions (Yu et al. 2014; Candeias et al. 2021; Zupancic et al. 2024). Even though a few studies also exist on element distribution between road dust and various non-biological aqueous solutions (Jayarathne et al. 2018; Kumar et al. 2020), our knowledge on the potential mobilization or retardation of contaminants in the environment and in the human body is still very limited.

Given these knowledge gaps, the study presented here had the overall objective to gain insights into potential interactions between road dust and both the environment and the human body. To achieve this goal, we conducted time-series leaching experiments with Philadelphia road dust, which was exposed to Gamble's solution (simulated lung fluid) and simulated gastric fluid, mimicking inhalation and ingestion, respectively. In addition, leaching experiments were carried out with synthetic rainwater and by following the EPA 3050B method. Therefore, our study not only provides new data on the leaching behavior of road dust over time, but also on the distribution, or partitioning, of chemical elements between road dust and different types of fluids, thus delivering quantitative data on both environmental availability and bioaccessibility of various chemical elements. Moreover, these experiments were carried out to test our hypothesis that greater concentrations of leached elements in the fluids are associated with lower pH values. This investigation further aimed at comparing mineralogical and chemical data for road dust to those obtained by O’Shea et al. (2020), as six of the same locations with high concentrations of heavy metals were used for sample collection.

## Materials and methods

Samples from six locations in Philadelphia were characterized using a multi-analytical approach based on Vigliaturo et al. (2018), O'Shea et al. (2020), and O'Shea et al. (2021a). It involved the methods of X-ray fluorescence (XRF), powder X-ray diffraction (XRD), and inductively coupled plasma optical emission spectroscopy (ICP-OES), as well as batch reactor/dissolution experiments.

### Sample collection

For this study, road dust was collected at six sites in the Philadelphia area (Supplementary Information; Table S1, Fig. S1), which were selected based on the varying Pb concentration levels reported in O’Shea et al. (2020). The sample ID numbers used here correspond to those of O’Shea et al. (2020). Geographically, the sites are located in the Center City and University of Pennsylvania campus areas. Based on the average annual daily traffic (AADT) data, three sampling locations are classified as medium-traffic (AADT = 6000–13,000), the other three as high-traffic (AADT > 13,000) sites (O’Shea et al. 2020).

The samples were collected between 4:00 a.m. and 6:00 a.m. in late July – early October 2021 using a handheld bag-less vacuum (BLACK + DECKER dust buster) held at street level for around 10–15 min at each site. The collection was performed 3–7 days after the last rain event and covered the entire width of the road. However, it was primarily focused on the edge as that is where the road dust is typically accumulated. Immediately after collection, samples were transferred into anti-static polyethylene bags and sealed. Prior to sampling at the next location, every part of the handheld vacuum was cleaned by washing and air-drying to avoid cross-contamination.

### Sieving

Each sample was dry-sieved using an 841-µm stainless-steel sieve employing regular, circular motions. The 841-µm sieve (ASTM No. 20 mesh) was chosen because road- dust particles are typically smaller than 850 µm across (Duong and Lee 2011) and it permitted for removal of coarser debris, including litter. Subsequently, a finer sieve (No.140 mesh, < 75 µm) was used to produce a size fraction that is more relevant to assess lung hazard. The weight of each sample was recorded both before and after sieving.

### XRF and XRD

In a first step, total bulk elemental content of each sieved coarse sample (< 841 µm) was determined via XRF (Vanta M, Olympus Corp.). These data helped in identifying elements of interest for subsequent analysis by ICP-OES and statistical interpretation. For each road-dust sample, five separate subsamples were analyzed; the mean and median were calculated from these five replicate measurements to account for within-sample heterogeneity (Supplementary Information; Table S2).

For the XRD analysis, the samples were crushed and powdered by hand for four minutes using an agate pestle and mortar. Subsequently, each sample was back-packed into 26-mm diameter specimen holders. A Panalytical X'Pert Pro X-ray diffractometer, equipped with a Co-K_α_ radiation source, was used to record the XRD pattern for each sample between 5º and 110° 2θ at 40 kV and 40 mA. The Panalytical software program HighScore Plus was then used to identify the major mineral phases present.

### Batch reactor experiments and ICP-OES

For the bulk chemical analysis of the road-dust samples, a hydrochloric/nitric acid digest, based on EPA 3050B Sect. 7.5 (United States Environmental Protection Agency [USEPA] 1996), was used to extract elements that might become environmentally available. Approximately 0.25 g of each sample was combined with 10 mL of the acid mixture and then heated to 95 °C in a graphite block digestor. The sample remained at these conditions for 30–45 min. Subsequent to the digestion, de-ionized water was added to each sample for a final volume of 50 mL, which were then filtered and analyzed by using ICP-OES (Genesis, Spectro GmbH) for the following pre-selected elements of interest: Al, Fe, V, Cr, Ni, Cu, Zn, Pb, As, and Cd. Six calibration standards (100 ppm, 50 ppm, 25 ppm, 10 ppm, 5 ppm, and zero ppm [blank]) were created for each element. For convenience, these elements were classified into major (Al, Fe) and minor elements (V, Cr, Ni, Cu, Zn, Pb, As, Cd), based on their general abundance in the Earth’s crust (Rudnick and Gao 2003). The limit of quantitation (LOQ) for these elements was (in ppm): Al = 0.03; Fe = 0.001; V = 0.002; Cr = 0.001; Ni = 0.004; Cu = 0.003; Zn = 0.0006; Pb = 0.015; As = 0.01; and Cd = 0.0005.

In order to imitate the behavior of the studied road-dust samples in the environment and in the human body, they were subjected to leaching experiments carried out with synthetic rainwater (ERM-CA408), simulated gastric fluid, and modified Gamble's solution (simulated lung fluid). The compositions for rainwater and gastric fluid were identical to those used by O'Shea et al. (2021b); Gamble’s solution was based on Vigliaturo et al. (2018). The *synthetic rainwater* contained the following constituents (concentrations in mg·L^−1^): NaF – 0.411; Na_2_SO_4_ – 2.019; NH_4_Cl – 2.569; Ca(NO_3_)_2_·4H_2_O – 1.817; Mg(NO_3_)_2_·6H_2_O – 1.636; NaNO_3_ – 0.381; KH_2_PO_4_ – 0.523; and NH_4_H_2_PO_4_ – 0.855; pH was 5.6, measured 24 h after the solution was made. The simulated *gastric fluid* was composed of 0.4 M glycine adjusted to pH 1.5 with ACS-grade HCl. *Gamble’s solution* consisted of the following components (concentration in g·L^−1^): MgCl·6H_2_O – 0.205; NaCl – 6.022; KCl – 0.299; Na_2_HPO_4_ – 0.126; Na_2_SO_4_ – 0.062; CaCl_2_·2H_2_O – 0.368; NaC_2_H_3_O_2_·3H_2_O – 0.953; NaHCO_3_ – 2.604; and Na_3_C_6_H_5_O_7_·2H_2_O – 0.098; pH was 8.0. Immediately after being mixed, both the simulated gastric fluid and the Gamble’s solution were stored overnight in an incubator at 37 °C until the beginning of the experiments, whereas synthetic rainwater was kept in the laboratory at *ca.* 21 °C. All experiments with the simulated biological fluids were carried out at 37 ºC to mimic human body conditions, whereas those with synthetic rainwater were performed at ~ 21 ºC to imitate ambient conditions.

For the synthetic rainwater experiment, approximately 1 g of the < 841 µm sieved fraction was added to 50 mL of the solution for each experiment. For the simulated gastric experiment, around 0.5 g of the < 841 µm fraction was added to 25 mL, whereas for the Gamble’s solution, approximately 0.3 g of the < 75 fraction µm was added to 25 mL of the fluid. The batch reactors were harvested at the following time points: 0, 0.5, 1, 12, 24, and 48 h (synthetic rainwater); 0, 1, 4, 8, 12, and 24 h (gastric fluid); and 0, 0.5, 1, 2, 4, 8, 12, and 24 h (Gamble's solution). Each experiment included two blank samples, in which no road dust was added, to test for impurities in the chemicals used to create the solutions and any contamination. At each time point, every harvested batch reactor was filtered using a 0.45 µm nylon membrane filter. All extractions were performed in triplicate. Collected supernatant harvests were then analyzed via ICP-OES (Genesis, Spectro GmbH) for the same elements determined in the original road dust.

The ICP-OES data were then processed with Microsoft Excel. Measured values were corrected for the mean blank value for each solution. The resulting value was multiplied by the volume of extractant used and divided by the subsample weight to get the final value of concentration extracted for each road-dust sample. All 0.5 values were rounded to the nearest even number.

## Results

### Road-dust samples prior to extraction

By far the highest overall elemental concentrations in the road dust, as determined by XRF (Table 1), were found for Al and Fe. The median and the mean values for the six samples of road dust were identical for each of these two elements (24,000 ppm for Al; 41,000 ppm for Fe). Of note are further the high mean contents of Zn (830 ppm), and the elevated mean concentrations of Cu (190 ppm), Cr (170 ppm), and V (130 ppm). Both Ni and Pb are present in moderately low concentrations (means of 38 and 69 ppm, respectively), but Pb contents as high as ~ 120 ppm were observed (sample 6; see Supplementary Information, Table S2).

**Table 1:** Descriptive statistics for the concentrations of the selected elements (in ppm and as determined by XRF) in all six samples of the Philadelphia Road Dust (coarse fraction)

Element	Min	Median	Max	Mean	SD^1^	SE^1^	IQR^1^	Kurtosis	Skewness
Al	20,000	24,000	27,000	24,000	2,600	1,100	2,900	−0.7	−0.4
Fe	34,000	41,000	48,000	41,000	6,100	2,500	11,000	−2.8	0.05
V	< 1	120	251	130	81	33	39	1.8	−0.06
Cr	53	180	270	170	75	31	64	0.8	−0.4
Ni	30	39	44	38	7	2.9	13	−3.0	−0.1
Cu	130	180	250	190	42	17	45	−0.3	−0.04
Zn	310	950	1,300	830	350	144	350	−0.5	−0.6
Pb	21	71	120	69	35	14	32	0.4	0.2
As	2	5	7	5	2	0.8	3	−1.4	−0.3
Cd	< 0.1	0.1	6	2	3	1.1	3	−0.4	1.2

^**1**^ SD = standard deviation; SE = standard error; IQR = interquartile range

The XRD patterns revealed that quartz and dolomite occur in all samples, representing the most prevalent mineral constituents of the studied road dust (Supplementary Information; Figs. S2 – S7). In addition, six other phases were detected, but they do not occur at each site: calcite, andesine, albite, anorthoclase, micas, and clay minerals (Supplementary Information; Table S1).

### Extraction results

#### EPA 3050B

In the EPA 3050B experiment, the concentration of each element released from the finer fraction (< 75 µm) of a given sample was generally higher than that of the coarse fraction, which was < 841 µm and thus also contained the < 75 µm fraction.

Of all six samples, road dust from Site 1 released the highest amount of Al, with 4,800 ppm from the coarse and 6,700 ppm from the fine fractions (Supplementary Information; Table S3). In contrast, road dust from Site 1 released the lowest amounts of Fe from both the coarse (8,100 ppm) and the fine (10,000 ppm) fractions. At most other sites, the released Fe concentrations were above 10,000 ppm, with the highest values observed for the samples from Site 6 (coarse: 18,000 ppm) and from Site 4 (fine: 17,000 ppm).

Of the minor elements, Zn was the predominant element extracted at all sites (Supplementary Information; Table S3), with the highest concentration found for Site 6 (coarse: 390 ppm, fine: 690 ppm) and the lowest for Site 1 (coarse: 93 ppm, fine: 150 ppm). Copper concentrations were second highest in the extracts, with the highest values observed for Site 4 (coarse: 230 ppm) and Site 5 (fine: 210 ppm), whereas the lowest concentrations for both fractions were seen for Site 1 (coarse: 51 ppm, fine: 90 ppm). For Pb, it was, again, Site 6 that had the highest (coarse: 68 ppm, fine: 140 ppm) and Site 1 the lowest (coarse: 9.2 ppm, fine: 19 ppm) concentrations. In the solutions from all sites, V and Cr were within the same concentration range, whereas Ni was slightly lower. The concentrations of As and Cd in the extracts were the lowest (all values < 3 ppm), with As not quantifiable in some samples, and consequently, these two elements will not be further discussed.

#### EPA 3050B vs. endpoint simulated environmental and bodily fluids extractions

**Fig. 1:**
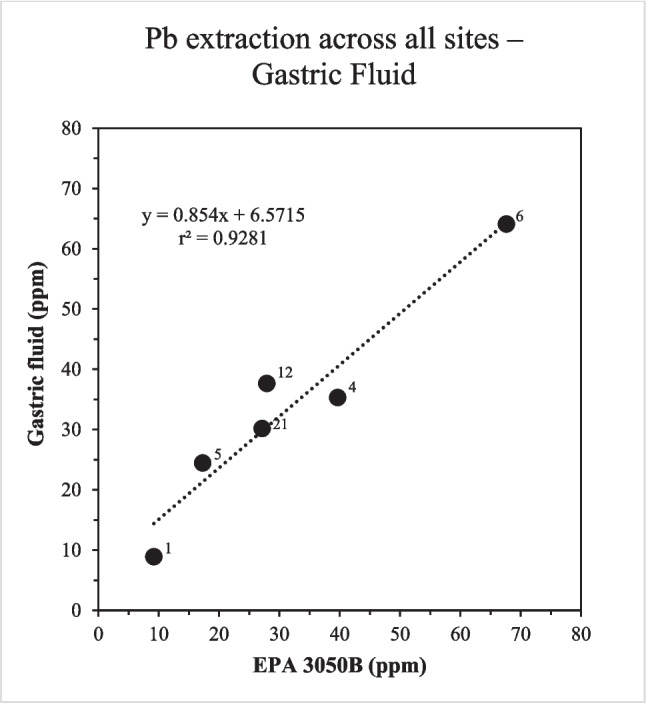
Comparison between the Pb concentrations in the extracts obtained using simulated gastric fluid (at final time point) and the Pb concentrations in the EPA 3050B extracts from the coarse fraction of the respective road dusts. Each dot represents a different site, identified by site numbers

The data revealed that, in general, the higher the concentration of any given element of interest extracted by EPA 3050B, the higher the amount extracted in the three test solutions. For example, in simulated gastric fluid, Pb showed a strong positive correlation (r^2^ > 0.9) with the data for the EPA 3050B experiments (Fig. 1). It further has a slope of 0.854, meaning that almost the same amounts of Pb were leached by the gastric fluid and the EPA 3050B extraction.

#### Pre-selected elements across different sites

At all sites, simulated gastric fluid was the most effective at elemental leaching amongst the three simulated fluids applied, followed by synthetic rainwater, and Gamble’s solution, in which almost no extraction was observed. Since our data indicated that each site was distinct, the results are presented per site (Tables 2, 3, 4, 5, 6 and 7), instead of per extraction fluid. These tables list the concentration determined for each site at the end point for the time-series of each dissolution experiment (48 h for synthetic rainwater; 24 h for both simulated gastric fluid and Gamble’s solution). These values can be compared to the *potential* environmental availability measured in the EPA 3050B solution (Table S3). The coarse fraction (< 841 µm) was used for the extraction experiments with synthetic rainwater and simulated gastric fluid, whereas the fine fraction (< 75 µm) was used for the experiments with Gamble’s solution, which is the fraction more likely to be inhaled.

**Table 2:** Site 1 – Concentrations measured at the final time point in the different solutions, reported in ppm. Values < LOQ are marked with “-”

Element	Synthetic Rainwater				Simulated Gastric Fluid				Gamble's Solution			
	Mean	SD	% Extr	SD	Mean	SD	% Extr	SD	Mean	SD	% Extr	SD
Al	20	13	0.08	0.05	3,200	170	12.5	0.8	-			
Fe	0.3	0.2	0.001	0.001	1,900	55	5.1	0.2	-			
V	0.004	0.006	0.002	0.004	2.10	0.08	0.8	0.1	1.06	0.03	0.42	0.05
Cr	0.14	0.09	0.3	0.2	2.2	0.2	4.2	0.8	-			
Ni	0.03	0.06	0.1	0.2	0.84	0.09	2.6	0.4	-			
Cu	0.4	0.4	0.3	0.3	35	9	28	11	-			
Zn	0.6	0.5	0.2	0.2	102	2	33	2	-			
Pb	0.04	0.07	0.2	0.3	9	1	43	6	-			

The percent extraction (% Extr.) values were calculated from the final concentration of each element in solution divided by their respective bulk concentration in the road dust (XRF; Table S2)

**Table 3:** Site 4 – Concentrations measured at the final time point in the different solutions, reported in ppm. Values < LOQ are marked with “-”

Element	Synthetic Rainwater				Simulated Gastric Fluid				Gamble's Solution			
	Mean	SD	% Extr	SD	Mean	SD	% Extr	SD	Mean	SD	% Extr	SD
Al	3	5	0.01	0.02	1,400	57	6.1	0.4	0.4	0.6	0.002	0.003
Fe	2	3	0.004	0.006	2,500	60	5.3	0.4	-			
V	0.002	0.004	0.001	0.003	1.61	0.08	1.0	0.2	-			
Cr	0.03	0.02	0.01	0.01	8	4	4	2	-			
Ni	0.02	0.01	0.05	0.03	3.2	0.5	7	2	-			
Cu	0.5	0.2	0.2	0.1	83	6	34	12	-			
Zn	1	2	0.1	0.2	310	30	31	6	-			
Pb	-				35	5	56	13	-			

The percent extraction (% Extr.) values were calculated from the final concentration of each element in solution divided by their respective bulk concentration in the road dust (XRF; Table S2)

**Table 4:** Site 5 – Concentrations measured at the final time point in the different solutions, reported in ppm. Values < LOQ are marked with “-”

Element	Synthetic Rainwater				Simulated Gastric Fluid				Gamble's Solution			
	Mean	SD	% Extr	SD	Mean	SD	% Extr	SD	Mean	SD	% Extr	SD
Al	3	4	0.01	0.02	1,200	93	5.3	0.4	-			
Fe	1	1	0.004	0.003	1,500	170	4.4	0.6	-			
V	0.01	0.01	0.01	0.01	1.3	0.1	1.0	0.2	-			
Cr	0.1	0.1	0.07	0.09	1.8	0.2	1.3	0.4	-			
Ni	0.004	0.007	0.01	0.03	1.1	0.3	4	1	-			
Cu	0.37	0.03	0.2	0.1	39	4	24	16	-			
Zn	0.6	0.5	0.1	0.1	190	55	36	12	-			
Pb	-				24	9	53	25	-			

The percent extraction (% Extr.) values were calculated from the final concentration of each element in solution divided by their respective bulk concentration in the road dust (XRF; Table S2)

**Table 5:** Site 6 – Concentrations measured at the final time point in the different solutions, reported in ppm. Values < LOQ are marked with “-”

Element	Synthetic Rainwater				Simulated Gastric Fluid				Gamble's Solution			
	Mean	SD	% Extr	SD	Mean	SD	% Extr	SD	Mean	SD	% Extr	SD
Al	0.6	0.4	0.002	0.001	1,300	65	5.1	0.3	0.2	0.3	0.001	0.001
Fe	1	2	0.003	0.004	1,700	110	3.6	0.3	-			
V	0.01	0.01	-		1.3	0.3	-		-			
Cr	0.018	0.007	0.01	0.01	4.1	0.6	2.2	0.7	-			
Ni	0.01	0.02	0.03	0.06	2.3	0.4	7	1	-			
Cu	0.5	0.1	0.23	0.09	87	26	40	16	-			
Zn	1	2	0.1	0.1	560	210	44	18	-			
Pb	-				65	23	53	21	-			

The percent extraction (% Extr.) values were calculated from the final concentration of each element in solution divided by their respective bulk concentration in the road dust (XRF; Table S2)

**Table 6:** Site 12 – Concentrations measured at the final time point in the different solutions, reported in ppm. Values < LOQ are marked with “-”

Element	Synthetic Rainwater				Simulated Gastric Fluid				Gamble's Solution			
	Mean	SD	% Extr	SD	Mean	SD	% Extr	SD	Mean	SD	% Extr	SD
Al	1.2	0.8	0.006	0.004	1,100	140	5	2	-			
Fe	5	3	0.015	0.008	1,700	200	4.9	0.6	-			
V	0.01	0.01	0.009	0.009	1.3	0.2	1.2	0.2	-			
Cr	0.05	0.01	0.03	0.01	4.0	0.4	2.3	0.7	-			
Ni	0.04	0.02	0.09	0.05	2.6	0.7	6	2	-			
Cu	0.6	0.5	0.3	0.3	63	16	32	23	0.4	0.3	0.2	0.2
Zn	8	3	0.8	0.3	290	27	31	6	-			
Pb	0.1	0.1	0.1	0.2	38	6	48	13	-			

The percent extraction (% Extr.) values were calculated from the final concentration of each element in solution divided by their respective bulk concentration in the road dust (XRF; Table S2)

**Table 7:** Site 21 – Concentrations measured at the final time point in the different solutions, reported in ppm. Values < LOQ are marked with “-”

Element	Synthetic Rainwater				Simulated Gastric Fluid				Gamble's Solution			
	Mean	SD	% Extr	SD	Mean	SD	% Extr	SD	Mean	SD	% Extr	SD
Al	1.0	0.3	0.004	0.001	1,200	170	4	1				
Fe	0.5	0.4	0.001	0.001	1,800	78	4.1	0.2				
V	-				0.8	0.2	0.7	0.2				
Cr	0.04	0.02	0.01	0.01	4	1	1.7	0.5				
Ni	-				2	1	5	3				
Cu	0.36	0.02	0.2	0.1	46	8	26	13				
Zn	0.003	0.005	0.0003	0.001	270	110	28	15				
Pb	-				30	6	36	10				

The percent extraction (% Extr.) values were calculated from the final concentration of each element in solution divided by their respective bulk concentration in the road dust (XRF; Table S2)

In this section, the leaching behavior of the eight elements after different time periods across different sites is presented.

##### Aluminum (Al)

**Fig. 2:**
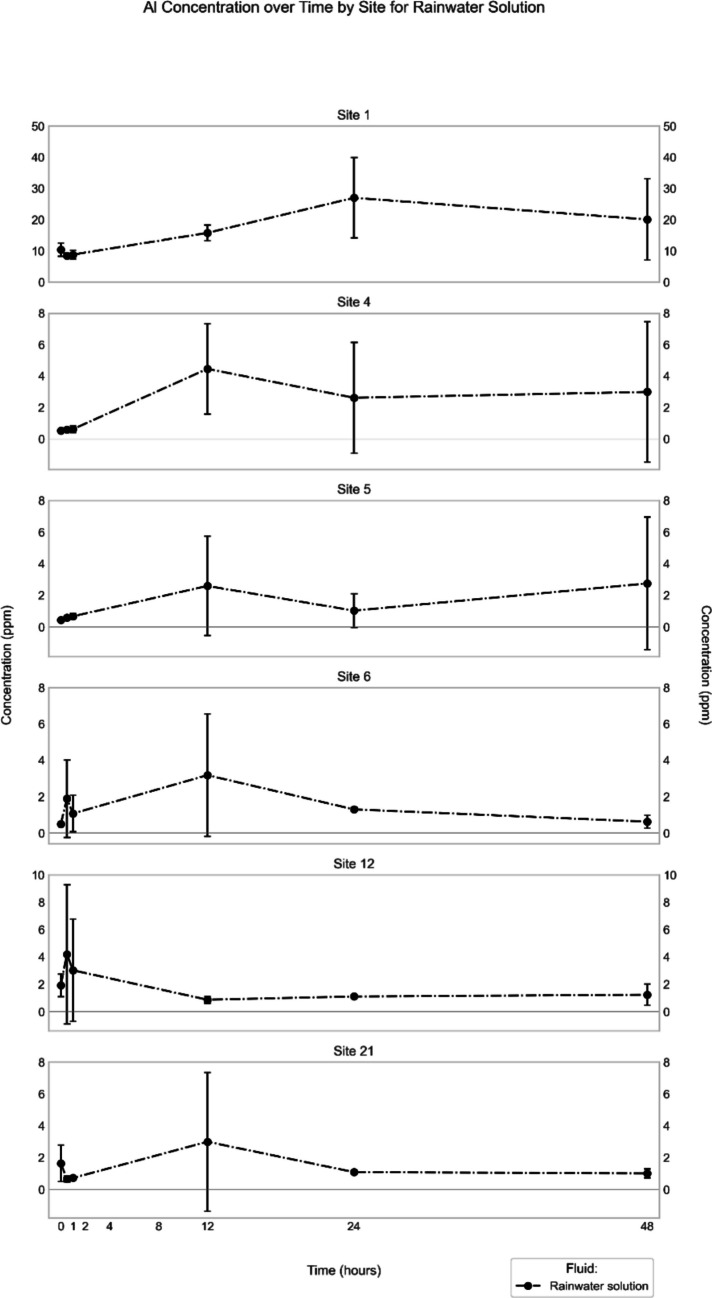
Mean (*n* = 3) Al concentration in synthetic rainwater extract (in ppm) through time for the different sites

**Fig. 3:**
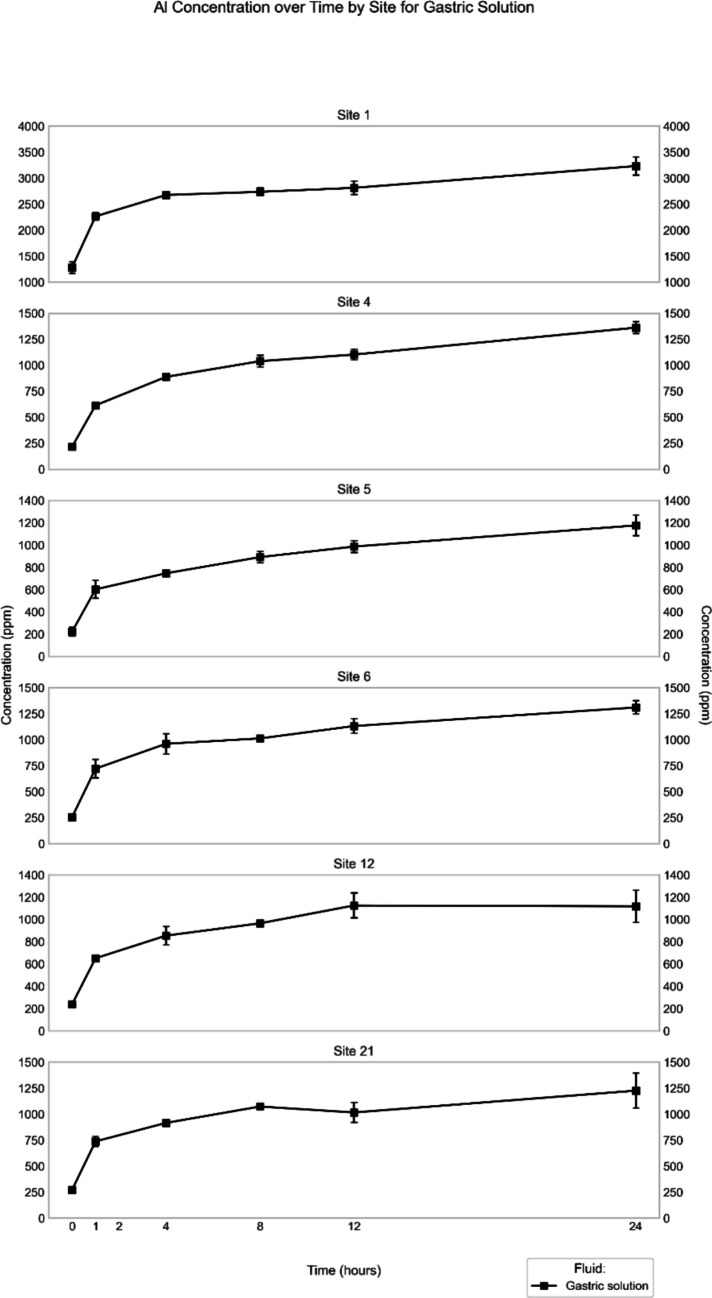
Mean (*n* = 3) Al concentration in simulated gastric fluid extract (in ppm) through time for the different sites

Of the six sampling locations, Site 1 showed the highest Al concentrations in the extracts of both synthetic rainwater and simulated gastric fluid (Figs. 2, 3). In synthetic rainwater, the maximum concentration was 27 ± 13 ppm, observed at the 24-h time point, whereas in the gastric fluid extract, the maximum concentration was much higher (3200 ± 170 ppm; Table 2), also recorded at the 24-h time point (end of experiment). Aluminum, however, was not quantifiable in the Gamble’s solution extract from Site 1 (Table 2).

**Fig. 4:**
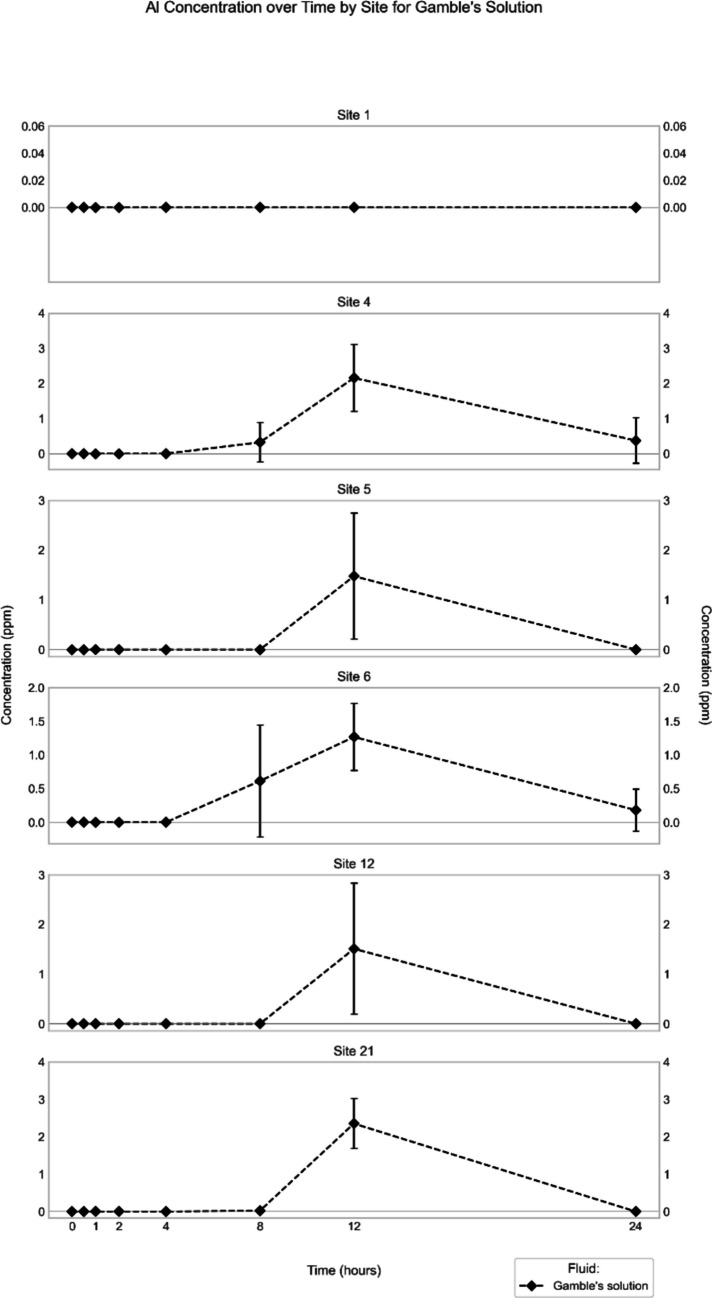
Mean (*n* = 3) Al concentration in Gamble’s solution extract (in ppm) through time for the different sites. Note that for Site 1, no quantifiable Al was extracted at any time point, in contrast to all other sites

The other sites showed patterns that were similar to each other. In synthetic rainwater, the Al concentrations peaked around the 12-h time point at 3–4 ppm (except for Site 12) but had high standard deviations (Fig. 2). In simulated gastric fluid, concentrations increased continuously until the 24-h end point, ranging between 1,100 and 1,400 ppm (Tables 3, 4, 5, 6 and 7). In contrast to Site 1, Al was quantifiable in the Gamble’s solution extracts from the other sites, with highest concentrations (1–3 ppm) recorded at the 12-h time point (Fig. 4).

It is also notable that Al was released immediately at all sites only in simulated gastric fluid (Fig. 3). On the other hand, it was only Sites 1, 6, 12, and 21 where extraction of Al was recorded in synthetic rainwater at time 0, whereas in the Gamble’s experiments, no sites released Al at time 0.

##### Iron (Fe)

The concentration of Fe in synthetic rainwater followed a pattern that was similar to that of Al in the same solution (Supplementary Information; Fig. S8). However, compared to Al, the concentration range of extracted Fe was lower (0.3–5 ppm), with the highest value observed for Site 12 at the end of the experiment.

In the simulated gastric experiment, the Fe concentrations and trends at all sites were similar to each other (Supplementary Information; Fig. S9). The extracts from Site 4, however, had slightly higher Fe concentrations at almost every time point. In general, Fe was released immediately (0-h time point) at all sites, and its concentration reached the highest values at the 24-h time point (1,500–2,500 ppm; Tables 2, 3, 4, 5, 6 and 7), again similar to the behavior of Al (Fig. 3).

The Fe concentration could not be quantified in the Gamble’s solution extract from any site at any time point (Tables 2, 3, 4, 5, 6 and 7).

##### Vanadium (V)

In synthetic rainwater, V was not extracted until the 12-h time point (Supplementary Information; Fig. S10). From there, its concentration then generally increased until reaching a peak at the 24-h time point, except for Sites 5, 6 and 12 whose peaks were recorded at the final time (48 h). At the final time point, Sites 5, 6, and 12 displayed the highest V concentrations (0.01 ± 0.01 ppm), whereas the final V concentrations in the other extracts were considerably lower, or even < LOQ (Tables 2, 3, 4, 5, 6 and 7).

In simulated gastric solution, V was released immediately (time 0 h) at several sites, from where its concentration increased gradually and steadily until the final time point (24 h) for all sites (Supplementary Information; Fig. S11). At this time point, the gastric fluid extract from Site 1 had the highest concentration (2.10 ± 0.08 ppm), whereas that from Site 21 had the lowest value (0.8 ± 0.2 ppm; Tables 2, 3, 4, 5, 6 and 7).

In Gamble’s solution, release of V was only recorded for Site 1 (Supplementary Information; Fig. S12). Here, the V concentration increased gradually and steadily to its highest value (1.06 ± 0.03 ppm), measured at the 24-h time point.

##### Chromium (Cr)

While no quantifiable Cr (< 0.001 ppm) was extracted during the experiment with Gamble’s solution, the element was released from all sites in both synthetic rainwater and simulated gastric fluid, even though the amounts released were very low (Supplementary Information; Figs. S13 and S14). In synthetic rainwater, Cr started to be released at all sites by the 12-h time point, whereby the highest concentrations were recorded for Site 1 at both the 24-h (0.3 ± 0.1 ppm) and the 48-h time (0.14 ± 0.09 ppm) points.

In simulated gastric fluid, Cr was released immediately at all sites, except Site 6, and reached its highest concentration after 24 h. The highest value of 8 ppm, albeit with a high standard deviation (± 4 ppm), was determined for Site 4 (Tables 2, 3, 4, 5, 6 and 7).

##### Nickel (Ni)

In synthetic rainwater, there was an immediate release of Ni only for Site 5; at the other sites, release started at the 12-h time point (Supplementary Information; Fig. S15). The sample from Site 12 released the most Ni, determined as 0.04 ± 0.02 ppm at the 48-h period.

In simulated gastric fluid, similar to the pattern seen for Cr, the highest Ni concentration was observed for Site 4, with a maximum and final value of 3.2 ± 0.5 ppm, measured at the 24-h time point (Supplementary Information; Fig. S16). The lowest amounts of Ni were determined in the Site 1 extracts, with a maximum value of 0.84 ± 0.09 ppm, observed at the 24-h time point as well. Other sites also exhibited maximum concentrations at the end of the time series (24 h), except for Site 6, for which a maximum concentration was observed at the 12-h time point, albeit with a large standard deviation (3 ± 1 ppm).

In Gamble’s solution, no Ni was released from any of the sites at any time point (< 0.004 ppm).

##### Copper (Cu)

During the synthetic rainwater and simulated gastric fluid experiments, all samples released Cu (Supplementary Information; Figs. S17, S18), whereas in Gamble’s solution, a significant release of Cu was only observed for Sites 5 and 12 (Supplementary Information; Fig. S19).

In synthetic rainwater, almost all concentrations peaked at the 24-h time point, whereby the extracts for Site 1 (1.1 ± 0.4 ppm) exhibited the highest value. However, the trends were different for road dust from Site 6, which continued to release Cu, reaching its highest concentration at the 48-h time point (0.5 ± 0.1 ppm), and from Site 12, which also had its peak at the 48-h time point (0.6 ± 0.5 ppm).

In the simulated gastric fluid, Cu was released immediately at time zero at all sites. The highest concentration was observed in the extract for Site 6 with 99 ± 50 ppm, measured at the 12-h time point. The Cu concentration in this extract generally increased, except for the 8-h timepoint, where a slight decrease in concentration was observed (56 ± 23 ppm). Also, for Site 21, the concentration peak (88 ± 80 ppm) was observed earlier (at 4-h time point).

In Gamble’s solution, the peak of the Cu concentration was generally observed at the 8-h time point, with mean values significantly > LOQ only for Sites 5 (1.2 ± 0.2 ppm) and 12 (3.1 ± 0.5 ppm; Supplementary Information; Fig. S19).

##### Zinc (Zn)

In synthetic rainwater, the extracts for Site 12 displayed the highest concentration of Zn, with 8 ± 3 ppm at the 48-h time point (Tables 2, 3, 4, 5, 6 and 7 and Supplementary Information; Fig. S20).

In simulated gastric fluid, the samples from Site 6 released the highest amounts of Zn, with 560 ± 210 ppm at the final time point (24 h; Supplementary Information; Fig. S21). Also, like the other elements in the simulated gastric solutions, Zn was released immediately at the beginning of the extraction.

In Gamble’s solution, samples from Site 12 only were observed to release Zn, with the highest concentration of 0.4 ± 0.1 ppm recorded at the 4-h time point (Supplementary Information; Fig. S22).

##### Lead (Pb)

**Fig. 5:**
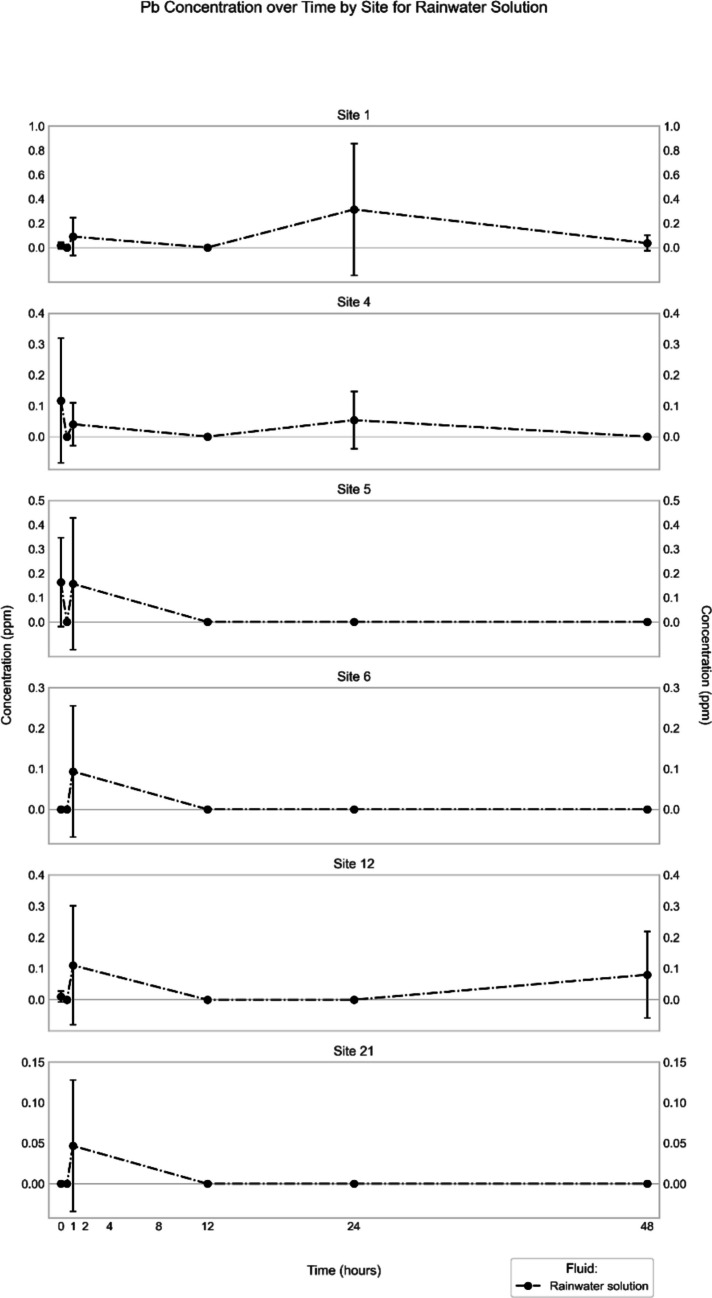
Mean (*n* = 3) Pb concentration in synthetic rainwater extract (in ppm) through time for the different sites

In the synthetic rainwater extracts of all sites, Pb concentrations were extremely low, typically < LOQ (Fig. 5). The highest concentration (0.3 ± 0.5 ppm) was found at Site 1, which was observed after 24 h from the beginning of the extraction. The highest Pb concentrations for the other sites were recorded immediately, or near the beginning of the extraction process (0- or 1-h time points), but they were < 0.2 ppm and associated with a high standard deviation.

**Fig. 6:**
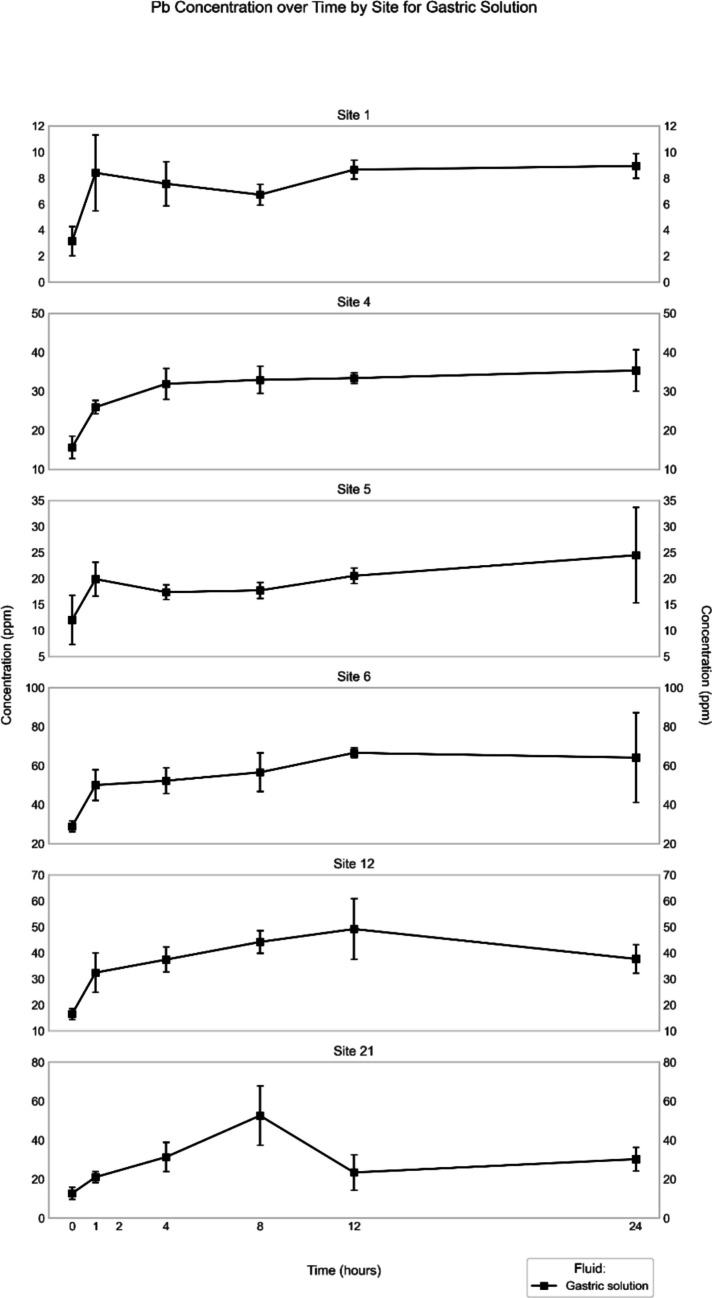
Mean (*n* = 3) Pb concentration in simulated gastric fluid extract (in ppm) through time for the different sites

In simulated gastric fluid, samples from Site 6 consistently released more Pb than those from the other sites, showing a peak concentration of 66 ± 2 ppm, observed at 12-h time point (Fig. 6) and a similar concentration, albeit with a relatively high standard deviation (65 ± 23 ppm) at the final time point. Similar trends are also seen for Sites 12 and 21, *i.e.*, their highest Pb concentrations were observed before the final time point with 49 ± 12 ppm (at 12-h period) and 52 ± 15 ppm (at 8-h period), respectively. Samples from the other sites (1, 4, and 5) generally continued to release Pb until the final time point (24 h), where concentrations of 9 ± 1 ppm, 35 ± 5 ppm, and 24 ± 9 ppm, respectively were reached.

In Gamble’s solution, no quantifiable Pb was released from any of the sites at any time point.

## Discussion

The results of this study provide new insights on four aspects of road dust: 1) Temporal variation of its bulk chemical composition; 2) Element extraction in different fluids; 3) Maximum element-extraction rates; and 4) Element distribution between fluids and road dust.

### Temporal variation of road-dust composition

O’Shea et al. (2020) examined road dust from the same six sites in Philadelphia that we studied here. Their material, collected in 2016, therefore provides an excellent opportunity to compare the bulk chemical composition of the road dust at two different points in time. The concentrations of both major and minor elements reported here are similar, with most concentration ranges overlapping, but our ranges are consistently narrower than those reported by O'Shea et al. (2020) (Supplementary Information; Table S4 and Fig. S23). For example, the Fe contents reported by O’Shea et al. (2020) ranged from 40,000 to 104,000 ppm (with a mean value of 65,000 ± 28,000 ppm), whereas the range we observed here for the same sites was 34,000 to 48,000 ppm, with a mean of 41,000 ± 6,100 ppm. Additionally, in O’Shea et al. (2020), the mean Pb concentration was 290 ± 240 ppm, with minimum and maximum values of 34 and 690 ppm, respectively, whereas in our study, the mean Pb concentration was 69 ± 35 ppm, with a minimum of 21 ppm and a maximum of 120 ppm. Similarly, the concentrations of Cu reported by O’Shea et al. (2020) were 460 ppm (mean), 130 ppm (min.), and 1200 ppm (max.), compared to 190 ppm (mean), 130 (min.), and 250 ppm (max.) determined in this study. The two data sets, thus, clearly demonstrate a temporal variability of the chemical composition of road dust at the study sites, which has also been documented previously in other cities. Variation of road-dust composition with time not only results from seasonal differences in atmospheric conditions (*e.g.*, summer *vs*. winter) but also from changes in the delivery and accumulation of natural materials (*e.g.*, soils) and in the sources of pollutants, such as vehicle speed, amount and composition of traffic, as well as construction and industrial activities (Lundberg et al. 2020; Švédová et al. 2020; Gustafsson et al. 2019; Dietrich et al. 2022; Coker et al. 2023).

On the other hand, the mineralogical composition of the road dust studied here is generally similar to what O’Shea et al. (2020) reported from the same locations, although magnetite and hematite were not detected in any of the samples investigated here. Quartz, the feldspar-group minerals, and the micas are likely derived from geologic sources, particularly the Wissahickon Formation, a mica-rich schist with abundant outcrops across the western parts of the city (purple areas in Fig. S1). Dolomite and calcite could have originated from the sidewalks, road surface, and/or construction materials. The clay minerals may be derived from the local soils or represent weathering products of micas and feldspars. The analytical technique used here (XRD), however, did not allow for identification of phases with very low abundances. Some of these non-detected phases might be anthropogenic and, at the same time, the predominant hosts for one or several of the chemical elements studied here, especially those related to industrial activities and not abundant in the minerals derived from the rocks of the Wissahickon Formation (*e.g.*, Cr, Ni, Cu, Zn, V, Pb; see O'Shea et al. 2020; 2021a). One such phase, for example, is crocoite, PbCr^6+^O_4_, which is still used as a pigment for yellow traffic paint at certain localities, including Philadelphia (O'Shea et al. 2021b).

### Element extraction in different fluids

The ICP-OES data for our leaching experiments revealed some general trends, which are very similar across the suite of samples and solutions used. At the same time, our data also highlighted some distinct element behaviors, which warrant special attention, because they point to differences in element speciation at different sites.

In general, our results document that as pH of the leaching solution decreases, larger amounts of the studied elements were extracted. The EPA 3050B method, a digestion based on a mixture of hydrochloric and nitric acid to assess *potential* environmental availability, extracted the largest amounts of the investigated elements. Simulated gastric fluid (pH = 1.5) came next (see Fig. 1), with a higher concentration of every element released compared to those determined in synthetic rainwater (pH = 5.6). Finally, Gamble’s solution (pH = 8.0) was least effective at extracting each of the investigated elements. Notably, most of the studied elements, including Pb and Cr, which can pose a health risk if present in a high concentration (Nag and Cummins 2022; Genchi et al. 2020, US EPA 2011, OSHA 1993), were not released at quantifiable levels during the interaction with Gamble’s solution.

**Fig. 7:**
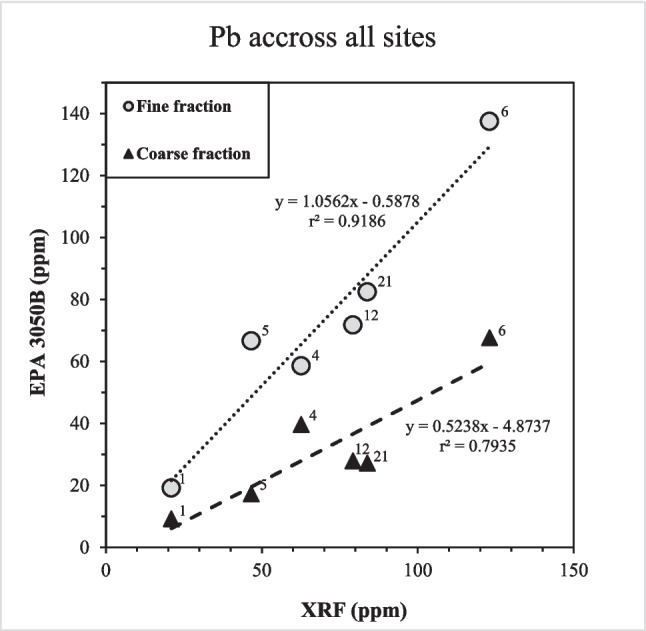
Mean (*n* = 6) bulk concentration of Pb in the road-dust samples, as determined by XRF, and Pb concentrations in extracts obtained by EPA 3050B for all sites

During the EPA 3050B extraction, the elemental concentrations released from the fine fractions (< 75 µm) were higher than those extracted from the respective coarse fractions (< 841 µm). This result was expected because finer materials have a higher specific surface area compared to that of the coarser fractions, allowing for enhanced reactivity. Moreover, when comparing the data obtained from the 3050B digest with those from XRF, a positive correlation was noted between these measurements, *i.e.*, in general, the higher the bulk concentration of a certain element in the road-dust samples, the more of that element was extracted by the 3050B method. An example of this relationship is provided by the Pb data, which reveal strong positive correlations for both the fine (*r*^2^ = 0.9186) and the coarse fractions (*r*^2^ = 0.7935; Fig. 7). This result implies that the higher the bulk Pb content of the road-dust sample is, the more Pb is potentially environmentally available. It further suggests that, while the coarse fraction only released around 50% of the total Pb present (slope of 0.5238; Fig. 7), Pb from the fine fraction was extracted completely (slope of 1.0562), which reflects the generally higher specific surface area of the finer material. Additionally, the complete removal of Pb from the fine fraction (< 75 µm), which is included in the coarse fraction (< 841 µm), also indicates that Pb was entirely present in the fine fraction. This conclusion, however, is based solely on the extraction behavior of Pb rather than on its directly measured concentration in the fine fraction of the road dust.

In the simulated *gastric-fluid* extracts, the major elements Al and Fe, as well as V showed a time-dependent increase in their concentration until they reached the final time point of the experimental observations. These general trends follow a logarithmic increase (Figs. 3, S9, S11), thus indicating that dissolution slowed down over time and that the concentration of these elements in the fluid might have reached a steady state at the end of the experiments. The trends, especially those for Al and Fe, are remarkably similar for all six studied samples of road dust, leading to excellent correlations (*r* ≥ 0.98) between the respective element concentrations at the various time points in the fluid (Figs. S24, S25). Excellent correlations are also observed between Al and V and between Fe and V in the road-dust leachates from all sites (Figs. S26, S27). These trends point to simultaneous release of the three elements from one specific solid phase present, most likely from the micas. In general, similar concentration–time trends are observed for the other minor elements in the gastric-fluid extracts as well, for example, Cr (Sites 6, 12, 21; Fig. S14), Ni (Site 4; Fig. S16), Cu (Sites 4, 12; Fig. S18), Zn (Sites 1, 4, 6; Fig. S21), and Pb (Sites 4, 6; Fig. 6). The correlations between the concentrations of these minor elements and those of Al or Fe, however, are less consistent across the sites (Fig. S24). To verify the hypothesis that many of the studied elements reached a near-saturation concentration in the gastric fluid, additional experiments would have to be performed over a longer time frame. For some minor elements, however, the logarithmic concentration increases are less well defined, or their concentration–time trends are even "undulating" at certain sites (*e.g.*, Pb for Site 21; Fig. 6).

In the synthetic *rainwater* extracts, the studied major and minor elements generally display poorly defined or undulating concentration–time trends, which in many cases record a maximum value somewhere along the experimental timeline rather than at the respective endpoints (Figs. 2, 5, S8, S10, S13, S15, S17, S20). One of the possible interpretations of such a trend is re-precipitation of the elements after reaching that maximum concentration. Alternatively, the apparently undulating patterns perhaps reflect some heterogeneity of the three subsamples used for each of the time points, which was indicated by a high standard deviation for some elements at some sites (Al at all sites, Fig. 2; Fe at Site 1, Fig. S8; Pb at all sites, Fig. 5; V at Sites 1, 12, 21, Fig. S10; Cr at Site 21, Fig. S13; Cu at Site 4, Fig. S17; Zn at Sites 1, 6, 21, Fig. S20). To validate whether re-precipitation happens, a more comprehensive and detailed experiment using a single reaction tube for all time points needs to be performed. Similarly, a scanning electron microscope investigation combined with energy-dispersive X-ray spectroscopy (SEM–EDX) of the solid materials, which accumulated at the bottom of the reaction tubes, would help in determining whether they contained any precipitates after the extraction. Some elements, however, display a continuous increase in concentration until the endpoint of the experiments (Site 6: Cr, Cu; Site 12: Fe, Zn), which indicates that extraction from the road dust would continue beyond the 48 h of exposure to rainwater, and that these elements might occur in a different speciation at these sites compared to the other ones. Another indication of speciation differences among the various sites is that the correlation matrices for the elemental concentrations are distinct (Fig. S28). For example, the concentrations of Cr, Fe, Zn and Pb show excellent correlations with each other at Site 1, but not at the other sites, or only for some of the elements (*e.g.*, Zn and Pb at Site 4). To verify the inferred differences in speciation of the elements at the various sites, however, additional analytical techniques would have to be employed (*e.g.*, sequential extraction, X-ray absorption near-edge structure [XANES] analysis), as has been done in other road-dust studies to determine the speciation of selected elements (Banerjee 2003; Lee et al. 2005, Barrett et al. 2010, Barrett et al. 2011).

In the *Gamble's solution* extracts, no general trends across the suite of samples are recognizable. Only a few elements, namely Al, V, Cu, and Zn, could be quantified, and only at some of the sites, documenting distinct behavior and pointing to different speciation. The road dust from Site 1, for example, is the only sample from which V was extracted by Gamble's solution, and the concentration–time trend shows a logarithmic increase, with release starting at about two hours of exposure (Fig. S12). At the same time, the road dust from Site 1 is the only one, from which no Al was extracted by Gamble's solution at quantifiable levels (Fig. 4). In all other cases, Al was released from the road dust, but not until approximately eight hours into the experiment. Moreover, all concentration–time trends display a maximum Al concentration at time point 12 h, from where the values decreased again (Fig. 4). The concentration–time-trends for Cu in Gamble's solution are similar for the samples from four of the six studied sites (Fig. S19), but only statistically documented for Sites 5 and 12. At these two sites, the concentrations of Cu start increasing after about two hours of exposure, reach a maximum at time point 8 h, and then decrease again later in the experiment (Fig. S19). Extraction of Zn by Gamble's solution was only indicated for Site 12, and the concentration–time trend is statistically not well defined (Fig. S22). As discussed above, undulating trends point to possible re-precipitation of certain phases, but the solids accumulated at the bottom of the reaction vessels would have to be investigated by SEM–EDX to test whether or not they might contain precipitates of Al, Cu, or Zn.

The experimental results further suggest that the road dust from Site 1, which has the highest bulk V contents among all sites (Table S2) and released the largest amounts of V into the gastric fluid (Fig. S11, Tables 2, 3, 4, 5, 6 and 7), hosts some of the V in a form that is more soluble in Gamble's solution than any of the other studied elements, possibly as a separate V-phase. In our XRD patterns, however, we did not observe a signal from such a phase, indicating that, if indeed present, its abundance would be very low. Our finding is consistent with previous experimental results, which documented that various simulated lung fluids, including Gamble's solution, were indeed able to extract V (as well as other metals) from fine particulate matter (PM) collected from urban air (Wiseman and Zereini 2014; Mazziotti Tagliani et al. 2017; Andrade-Oliva et al. 2025). The distinct V-phase postulated to occur in the road dust from Site 1 is most likely not present at the other sites, which in contrast, must contain an Al-phase that is soluble in Gamble's solution, possibly alumina (Al_2_O_3_). Alumina, specifically γ-Al_2_O_3_, has a low solubility in water, but as an amphoteric oxide, its solubility is pH-dependent, whereby it is higher in both acidic and basic conditions than in the neutral pH range (Driscoll and Schecher 1990; Baumgarten et al. 1995; Bojórquez-Quintal et al. 2017). More recently, however, it has been shown that nanoparticles of γ-Al_2_O_3_ are indeed soluble in Gamble's solution (pH = 8), and that its dissolved concentration increased with the time of exposure during experiments that lasted 24 h (Holmfred et al. 2022). Our results, thus, suggest that the road dust from Sites 4, 5, 6, 12, and 21 might contain small amounts of alumina, unlike the sample from Site 1. This phase, however, was not detected by XRD, and therefore, most of the Al in our samples would still be present in the Al-rich silicates (micas, clay minerals, feldspars) in the road dusts from all sites. To verify the presence of the postulated V- and Al-phases, the road- dust samples would have to be studied in detail by additional analytical methods, including SEM–EDX, transmission electron microscopy (TEM), and XANES analysis.

### Maximum element-extraction rates

To determine the maximum rate of element extraction (ppm/h) during the various leaching experiments, the maximum concentration of a given element determined for the extracting fluid was divided by the time at which that maximum was observed for each site. For example, in our experiments with synthetic rainwater, Al showed the highest maximum extraction rate for the road dust from Site 12 (8 ppm/h; Table 8), as its maximum concentration (4 ± 5 ppm) was reached at time point 0.5 h. Meanwhile, even though the highest maximum Al concentration (27 ± 13 ppm, Fig. 2) in synthetic rainwater among all sites was observed in the extracts from the Site-1 sample, it took 24 h to reach that point, and thus, the maximum extraction rate was only 1.1 ppm/h. Likewise, in the case of Pb, even though the sample from Site 1 released the highest amount of Pb (0.3 ± 0.5 ppm, Fig. 5), the maximum extraction rate (0.01 ppm/h) was lower than that of Site 12 (0.1 ppm/h), because 24 h were needed for the Site-1 solution to reach its maximum concentration, whereas it only took one hour in the case of Site 12.

**Table 8:** Maximum extraction rates in synthetic rainwater experiment, reported in ppm/hour. Asterisks refer to instantaneous concentrations, *i.e.,* concentrations at time 0 h

Site	Al	Fe	V	Cr	Ni	Cu	Zn	Pb
1	1.1	0.2	0.0002	0.012	0.002	0.04	0.05	0.01
4	0.3	0.1	0.0004	0.0013	0.0004	0.033	0.12	**
5	0.06	0.08	0.0002	0.002	*	0.022	0.038	***
6	0.2	0.02	0.0002	0.0004	0.0006	0.010	0.02	0.1
12	8	0.10	0.0002	0.0050	0.0008	0.012	0.17	0.1
21	0.2	0.2	0.0002	0.002	0.002	0.019	0.2	0.05

Measured mean concentrations at 0 h: * 0.019 ppm; ** 0.115 ppm; *** 0.165 ppm

We propose that these maximum element-extraction, or -release, rates may be viewed in connection with “residence time”, *i.e.*, the time that the road-dust samples were exposed to the environmental or bodily fluids, which could be related to how long the material can pose a risk. Road dust, for example, may be exposed to rainwater for a day or more, depending on the meteorological conditions, whereas in the gut system, after ingestion, it is typically exposed to gastric fluids for a more limited time only. According to the Mayo Clinic (n.d.), consumed food takes approximately 6–8 h to pass through stomach and small intestine, and then 36 h to complete the passage through the entire colon.

Our results revealed that the maximum extraction rates of nearly all studied elements were highest for the experiments with simulated gastric fluid and lowest for most of the elements in the experiments with Gamble's solution (Tables 8, 9 and 10), even though the material exposed to Gamble's solution was smaller (< 75 µm) than that used for the other two experiments (< 841 µm). At Site 21, for example, the maximum extraction rate of Al was 50 ppm/h in simulated gastric fluid, whereas it was only 0.20 ppm/h in Gamble’s solution. Another example is provided by Pb, whose maximum extraction rates in simulated gastric fluid were ≥ 1.0 ppm/h for all but one site, whereas those in synthetic rainwater were all ≤ 0.1 ppm/h; as noted above, no Pb was extracted by Gamble's solution from road dust at any site. On the other hand, the maximum extraction rate of V from Site-1 road dust in Gamble's solution (0.044 ppm/h) was two orders of magnitude higher than that observed for any site in synthetic rainwater and, moreover, it was similar in magnitude to that determined in simulated gastric fluid for all sites, where the highest value (0.088 ppm/h) was also recorded for Site 1. These data provide additional support for the conclusion that the road dust from Site 1 probably contains a separate V-phase, which does not occur at the other sites.

**Table 9:** Maximum extraction rates in simulated gastric fluid experiment, reported in ppm/hour

Site	Al	Fe	V	Cr	Ni	Cu	Zn	Pb
1	133	79	0.088	0.092	0.035	1.4	4.25	0.38
4	58	104	0.067	0.3	0.13	3.4	13	1.4
5	50	62	0.054	0.075	0.04	1.6	16	1.0
6	54	71	0.05	0.17	0.2	8	23	5.5
12	92	71	0.054	0.17	0.11	2.6	29	4.1
21	50	75	0.033	0.17	0.08	22	37	6

**Table 10:** Maximum extraction rates in Gamble’s solution experiment, reported in ppm/hour

Site	Al	Fe	V	Cr	Ni	Cu	Zn	Pb
1			0.044					
4	0.18					0.01		
5	0.1					0.15		
6	0.11							
12	0.2					0.39	0.10	
21	0.20					0.005		

A comparison of the bulk concentrations in road dust (XRF data, Table S2) with the calculated maximum extraction rates in rainwater (Table 8) reveals that a higher initial bulk element concentration in road dust generally corresponds to a higher maximum extraction rate, as observed, for example, for Pb (21 ppm, 0.01 ppm/h at Site 1; 79 ppm, 0.1 ppm/h at Site 12). However, this is not always the case in a site-by-site comparison. For example, the road dust from Site 1 contains ~ 25,000 ppm Al, whereas that from Site 12 has an Al content of ~ 20,000 ppm (Supplementary Information; Table S2), but the maximum Al-extraction rate from Site-1 road dust by synthetic rainwater was far lower than that recorded for Site 12. This result, in combination with the fact that Al was extractable by Gamble's solution from the Site-12 but not from the Site-1 sample, is supporting the conclusion that the road dust from Site 12 contains some Al in a different speciation.

In the gastric fluid experiments, a higher bulk concentration in the road dust is also associated with a higher maximum extraction rate for some of the studied elements, especially Al, Fe, and Pb (Table 9). For example, the mean bulk concentration of Pb in road dust from Sites 1, 5, and 6 was 21, 47, and 123 ppm, respectively, and the corresponding maximum extraction rates of Pb were 0.38, 1.0, and 5.5 ppm/h, respectively. Similar results are also observed for the Gamble’s solution experiments, even though only a few elements were released (Table 10). As an example, Al from Sites 4, 12, and 21 had a maximum extraction rate of 0.18, 0.2, and 0.20 ppm/h, whereas their bulk concentrations were ~ 23,000, ~ 20,000, and ~ 27,000 ppm, respectively.

The maximum extraction rates observed in the synthetic rainwater experiments suggest that road dust that is exposed to rainwater will release the studied elements relatively slowly. This conclusion implies that road dust will most likely release its components as long as it is lying on the road and exposed to rainwater, and consequently, until either the entire amount of that element has been released or until the road-dust particles have been swept away by road runoff. In the latter case, the particles will most likely continue to release their components, which will then be diluted if they end up in a surface water, such as a river. However, in the case of bodily fluids, which we used to simulate interaction of the road dust with lung and gastric fluids subsequent to its unintentional inhalation or ingestion, the risk differs significantly. The simulated gastric-fluid experiments showed a continuous release over a limited time, which is the critical time when the person is at greater risk. For inhaled particles, which are much smaller in size, the residence time is more difficult to predict, because they are deposited in different parts of the respiratory system depending on size: particles < 10 µm across (PM_10_) are trapped in the upper airway or conducting lower airways, whereas particles < 2.5 µm across (PM_2.5_) are deposited in the bronchioles and alveoli, where they will remain for very long periods of time (Oberdörster 1993; Kreyling et al. 1999; Möller et al. 2004; Kastury et al. 2017). Therefore, even if elements exhibit very small maximum extraction rates, they can be leached into the lung fluid.

### Element distribution between fluids and road dust

One of the most important methods used to estimate the mobilization and retardation potential of contaminants in the environment consists of experimentally determining the element distribution, or partitioning, between coexisting solid and aqueous phases (USEPA 2004; Kumar et al. 2020). Our data provide information on the element distributions between the bulk road dust and the fractions that are considered potentially environmentally available, environmentally available, or bioaccessible.

**Fig. 8:**
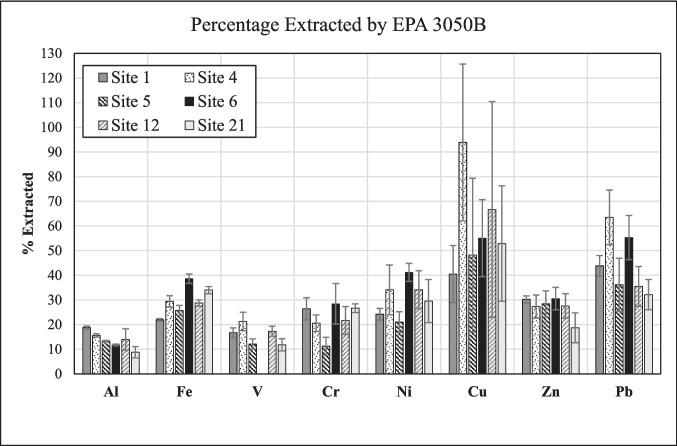
Element concentrations at each site, expressed as percentage of their concentration in the EPA 3050B extract relative to their bulk concentration in the respective road dust. All data obtained for the coarse fraction (< 841 µm). Shown error bars reflect concentration variabilities in the bulk road dust only, and therefore, represent a minimum estimate of the total uncertainty

To determine the potentially environmentally available element concentrations in the original road-dust samples we used the EPA 3050B method. Because this method is not a total digestion of the solid material (USEPA 1996), the obtained elemental concentrations differ from those determined by XRF for the same size fraction (see Tables S2, S3). The results revealed that the potentially environmentally available amounts of the studied elements vary greatly between the different metals and sites (Fig. 8). For example, depending on the site investigated, between 40 and 94% of the total amount of Cu present in the bulk road dust are potentially environmentally available (Sites 1 and 4, respectively), but the uncertainty on the percentage extracted is large (Table S5). On the other hand, much smaller amounts of V (≤ 21%) are potentially environmentally available at any of the sites (Table S5; Fig. 8). In regard to the major components, there are also marked differences in the distribution between the bulk road dust and the EPA 3050B leachate (Fig. 8). For example, the percentages of extracted Al and Fe at Site 1 (18.8 ± 0.6% and 21.9 ± 0.5%, respectively) are significantly different from those observed at Site 6 (11.7 ± 0.4% and 39 ± 2%; Table S5).

To assess the elemental distributions resulting from exposing the road dust to the other fluids, we used the final metal concentrations in the leachates, determined at the end of each experiment, because we assumed that the asymptotic approach over time to these final concentrations, observed in many cases (see above), points to an equilibrium state of the interaction (Julien et al. 2011; Mazziotti Tagliani et al. 2017). These final concentrations were then expressed as percentage of the extracted elements relative to their bulk concentrations in the original road dust (Table S2). The results thus describe the element distribution as leachability or environmental availability, and in the case of the simulated biological fluids, as bioaccessibility. Similar approaches have been taken in studies of elemental distribution between fine atmospheric PM and simulated lung fluids (Julien et al. 2011; Mazziotti Tagliani et al. 2017).

**Fig. 9:**
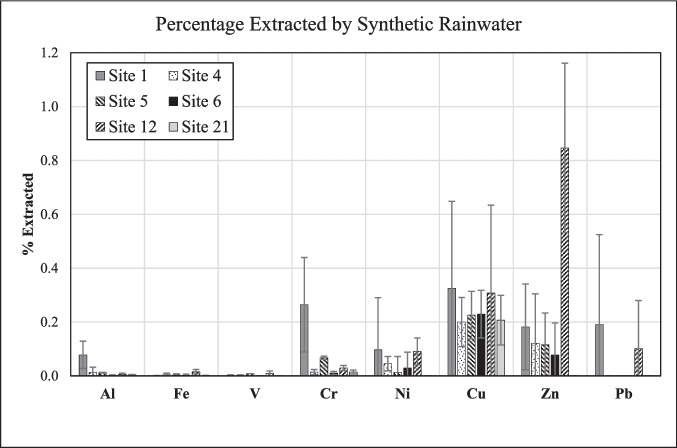
Element concentrations at each site, expressed as percentage of their final concentration in synthetic rainwater relative to their bulk concentration in the respective road dust. All data obtained from the coarse fraction (< 841 µm) of the road-dust material

The element distribution determined on the basis of leaching the road-dust samples with *synthetic rainwater* represents another, and in our opinion, more realistic approach to assessing environmental availability of metals than the one based on the EPA 3050B method. Compared to the EPA 3050B method (Fig. 8), leaching of the road-dust samples with synthetic rainwater was markedly less effective. Relative to the bulk road dust, all elements were extracted at < 1% (Fig. 9; Tables 2, 3, 4, 5, 6 and 7). This result suggests that, when road dust in an urban setting is exposed to rainwater for up to 48 h, it will only release relatively small amounts of the investigated metals. The data, however, also highlight that there are distinct differences between the various sites. For example, the data reveal that considerably higher percentages of Al, Cr, and Pb were extracted from the road dust at Site 1 than from the material at any other site. On the other hand, the highest percentage of extracted Zn is seen for Site 12 (0.8 ± 0.3%), significantly higher than for any other site; for instance, almost no Zn was extracted from the Site-21 material (Fig. 9). The observed differences in element distributions at the various locations support our conclusion that there are differences in metal speciation among the investigated road dusts.

**Fig. 10:**
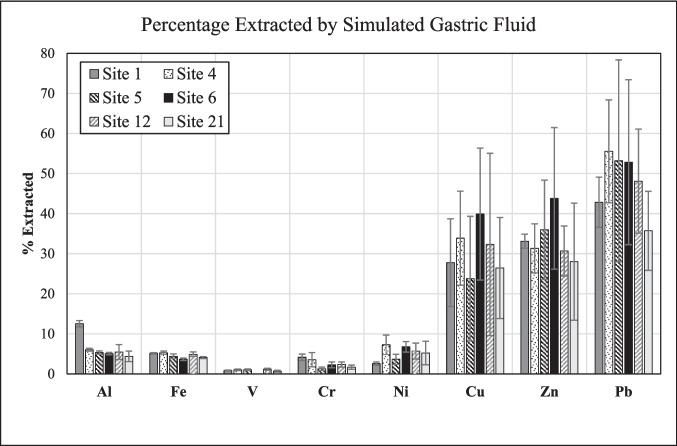
Element concentrations at each site, expressed as percentage of their final concentration in simulated gastric fluid relative to their bulk concentration in the respective road dust. All data obtained from the coarse fraction (< 841 µm) of the road-dust material

The element distribution between the leachates obtained by exposing road dust to *simulated gastric fluid* and the bulk road dust also reveals considerable differences between diverse metals and sites. Site 1, for example, exhibits a markedly higher percentage of extracted, and thus bioaccessible, Al than all other sites (Fig. 10), like in the case of synthetic rainwater (Fig. 9). Another striking feature seen in Fig. 10 is that the percentages of extracted Cu, Zn, and Pb are markedly and significantly higher than those of the other metals, even when considering the high standard deviations. In fact, the distribution of Pb between the bulk road dust and the simulated gastric fluid at all sites is remarkably similar to that seen between the bulk road dust and the EPA3050B extract (*cf.* Figures 8 and 10). On the other hand, Zn tends to be partitioned more strongly into the simulated gastric fluid (28–44%) than into the EPA 3050B extract (19–30%; Figs. 8, 10). In general, leaching of the road dust by simulated gastric fluid was more effective than by synthetic rainwater, but – except for Zn – less effective than leaching by the EPA 3050B method.

**Fig. 11:**
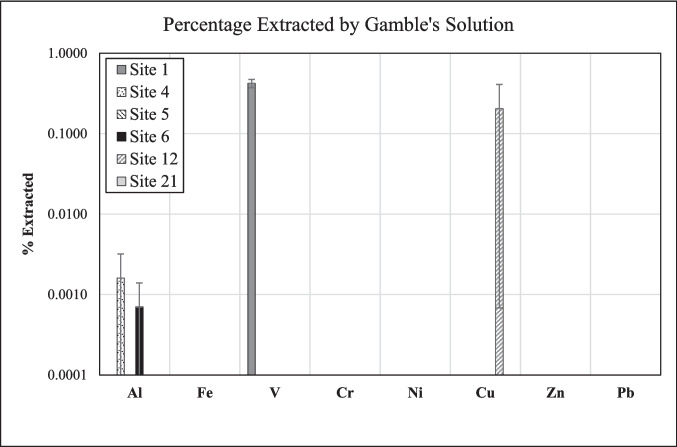
Element concentrations at each site, expressed as percentage of their final concentration in Gamble's solution relative to their bulk concentration in the respective road dust (< 841 µm). The material exposed to Gamble’s solution, however, was the fine fraction (< 75 µm) of the road dust

Only some metals (Al, V, Cu) and only at some of the sites were bioaccessible after the fine fraction of the road dust had been exposed to *Gamble's solution* for 24 h. The extracted amounts of these metals, however, were very small (Fig. 11). For example, less than 0.01% of the bulk Al in the road dust was bioaccessible, and only in the material from Sites 4 and 6 (Fig. 11). The percentages of extracted Al, however, are associated with large uncertainties (Tables 3,5). Similarly, V was released from Site-1 road dust only, and the percentage extracted was 0.42 ± 0.05%. This value, however, was significantly higher than that observed for synthetic rainwater (0.002 ± 0.004%; Table 2). This discrepancy is probably due to the fact that the percentages were calculated relative to the bulk road dust (< 841 µm), because we did not have equivalent XRF data for the fine fraction (< 75 µm), which was used for all experiments with Gamble's solution. Indeed, the fine fraction of the road dust at Site 1 is richer in V than the bulk material, as indicated by the amounts of potentially environmentally available V present (61 *vs.* 42 ppm, according to the EPA 3050B method; Table S3). A similar situation also exists for Cu at Site 12, the only site where the element was quantifiable after interaction of the road dust with Gamble's solution (Fig. 11). The percentage of extracted Cu, however, was low (0.2 ± 0.2%) but similar to that observed for synthetic rainwater (0.3 ± 0.3%; Table 6), which again most likely reflects the higher concentration of potentially environmentally available Cu in the fine fraction compared to that in the bulk material (190 *vs.* 130 ppm; Table S3). Overall, the observed element distributions between road dust and Gamble's solution provide further support to our conclusion that differences in the speciation of these metals in the road dust must exist between the studied sites.

## Conclusions

This experimental investigation on the dissolution of road dust provides new insights on how this material reacts with simulated environmental and bodily fluids and further presents an alternative assessment of how long the material may pose a risk to humans and the environment.

The road dust studied here exhibits ranges in metal concentrations that are consistently narrower than those reported for the analogous material collected in 2016 at the same locations, documenting a temporal variation of its bulk composition. Moreover, our extraction experiments revealed that, in general, the more acidic the solution was the higher its leaching capacity. In addition, we observed a positive correlation between the initial elemental concentration of a given element in the bulk road dust and its potential environmental availability. Moreover, our data document that the finer size fraction released higher amounts of elements than the coarser fraction, most likely due to its higher specific surface area.

*Simulated gastric fluid* leached relatively high amounts of metals, especially Cu, Zn, and Pb, from the road dust, indicating a potential health risk through ingestion. The logarithmic increase of the elemental concentrations with time, observed for Al, Fe, and several minor elements, suggests that a steady state has been achieved by the end of the experiment (24 h). *Synthetic rainwater* extracted relatively small amounts of metals, suggesting low mobilization under typical environmental conditions. The concentration–time trends are logarithmic only for a few elements at some sites; in several cases, however, the trends exhibit a maximum before the endpoint of the experiments, which we interpret as resulting from re-precipitation of the elements as some secondary phase. Other concentration–time trends in synthetic rainwater are undulating, which reflects some heterogeneity of the three subsamples used for each of the time points, as also indicated by a high standard deviation for the concentration of some elements at some sites. In *Gamble’s solution*, only small amounts of certain metals (Al, V, Cu, Zn) were extracted, but not from all materials.

Our results demonstrate that potential environmental availability, environmental availability, and bioaccessibility of metals in the studied road-dust samples vary considerably by element and location, which points to differences in the mineralogical composition of the materials collected from different sites. In particular, we interpret the distinct leaching behaviors of Al and V in Gamble's solution as resulting from the presence of minor Al- and V-phases at some of the sites. Such phases, however, have not been directly observed by XRD. The results further document that it is difficult to draw general conclusions about possible environmental and health impacts of road dust, unless the speciation of potentially toxic elements has been determined. Therefore, we recommend that future studies consider additional analytical methods, such as SEM–EDX, TEM, and XANES, to more fully characterize both the original road dust and the solids that remain or accumulate in the test tubes after exposure to the extracting fluids, as well as to further investigate the effects of speciation on the release of potentially toxic elements present in road dust. In addition, we propose that the maximum element-extraction rate, as defined in our study, is a helpful tool to evaluate effects of speciation on leaching behavior and duration as well as on potentially associated health risks subsequent to unintentional ingestion or inhalation of road-dust particles.

## Supplementary Information

Below is the link to the electronic supplementary material.

Supplementary file1 (DOCX 3.46 MB)

## Data Availability

The data that supports the findings of this study are available from the corresponding author upon request.
