# Supplementary material for: Experimental dissolution of road dust in simulated environmental and biological fluids
Source: Environ Sci Pollut Res Int. 2026 May 7;33(16):7812–35. doi: 10.1007/s11356-026-37727-7 (PMC13190782; doi:10.1007/s11356-026-37727-7)
Supplement: Supplementary file 1 — Supplementary file1 (DOCX 3.46 MB) [file 11356_2026_37727_MOESM1_ESM.docx]

**SUPPLEMENTARY INFORMATION FOR**

**Experimental dissolution of road dust in simulated environmental and biological fluids**

Ahmad Kamal Mubarok^1*^ and Reto Gieré^1,2^

* Corresponding author: ahmadkamalmu6@gmail.com / +1-267-4669075 (mobile)

^1^  Department of Earth and Environmental Science, University of Pennsylvania, Philadelphia, PA 19104, United States

^2^  Center of Excellence of Environmental Toxicology, University of Pennsylvania, Philadelphia 19104, PA, United States

**Table S1.** Sample locations and mineral phases (identified by XRD) at each site.

| **Name** | **Location & site description** | **Latitude** | **Longitude** | **Mineral phases** |
| --- | --- | --- | --- | --- |
| **Site 1** | 5th & Market St, near Liberty Park | 39.950459 | -75.148089 | Quartz, dolomite, calcite, andesine, albite, mica/ clay minerals |
| **Site 4** | 37th St & Locust Walk, university/city road | 39.952216 | -75.199178 | Quartz, dolomite, anorthoclase, calcite, mica/ clay minerals |
| **Site 5** | Between Hayden Hall & Shoemaker Green, university road | 39.951284 | -75.190590 | Quartz, dolomite, calcite, albite, mica/ clay minerals |
| **Site 6** | 30th & Arch St (in front of Cira Center), near main transport station - off highway | 39.957017 | -75.181871 | Quartz, dolomite, albite, mica/ clay minerals |
| **Site 12** | 15th & Arch St, Central Philadelphia Road | 39.954645 | -75.165146 | Quartz, dolomite, albite, mica/ clay minerals |
| **Site 21** | Walnut Bridge (over Schuylkill River) | 39.951538 | -75.181789 | Quartz, dolomite, calcite, anorthoclase, mica/ clay minerals |

**Table S2.** Mean elemental concentrations (*n* = 5) in the bulk (<841 µm) road-dust samples, as determined by XRF. All data reported as parts per million (ppm). SD = Standard Deviation. The first eight entries are the elements of interest in this study.

| **Element** | **Site 1** | |  | **Site 4** | |  | **Site 5** | |  | **Site 6** | |  | **Site 12** | |  | **Site 21** | |
| --- | --- | --- | --- | --- | --- | --- | --- | --- | --- | --- | --- | --- | --- | --- | --- | --- | --- |
|  | **Mean** | **SD** |  | **Mean** | **SD** |  | **Mean** | **SD** |  | **Mean** | **SD** |  | **Mean** | **SD** |  | **Mean** | **SD** |
| **Al** | 25,555 | 821 |  | 23,113 | 1,007 |  | 22,544 | 426 |  | 25,644 | 804 |  | 20,119 | 6,372 |  | 27,380 | 7,265 |
| **Fe** | 36,922 | 884 |  | 47,584 | 3,712 |  | 34,220 | 2,759 |  | 46,619 | 2,303 |  | 34,793 | 1,562 |  | 44,130 | 1,884 |
| **V** | 251 | 29 |  | 160 | 28 |  | 124 | 21 |  | < 1 |  |  | 110 | 13 |  | 119 | 25 |
| **Cr** | 53 | 9 |  | 214 | 35 |  | 134 | 44 |  | 183 | 53 |  | 171 | 45 |  | 274 | 18 |
| **Ni** | 31 | 3 |  | 44 | 13 |  | 30 | 6 |  | 34 | 3 |  | 44 | 10 |  | 44 | 13 |
| **Cu** | 126 | 36 |  | 245 | 83 |  | 164 | 106 |  | 218 | 62 |  | 195 | 128 |  | 174 | 77 |
| **Zn** | 308 | 15 |  | 989 | 168 |  | 528 | 98 |  | 1278 | 190 |  | 945 | 171 |  | 964 | 314 |
| **Pb** | 21 | 2 |  | 63 | 11 |  | 47 | 14 |  | 123 | 20 |  | 79 | 18 |  | 84 | 16 |
| **As** | 2 | 2 |  | 7 | 2 |  | 5 | 1 |  | 7 | 4 |  | 5 | 3 |  | 3 | 6 |
| **Cd** | 6 | 8 |  | < 0.1 |  |  | 4 | 10 |  | < 0.1 |  |  | < 0.1 |  |  | < 0.1 |  |
| **Ca** | 80,330 | 2,417 |  | 51,573 | 3,798 |  | 48,550 | 3,040 |  | 51,282 | 1,358 |  | 63,409 | 2,776 |  | 56,807 | 3,069 |
| **Mg** | 13,791 | 495 |  | 13,950 | 720 |  | 10,681 | 530 |  | 11,876 | 431 |  | 13,782 | 1,522 |  | 12,836 | 1,124 |
| **K** | 5,389 | 165 |  | 6,128 | 390 |  | 6,232 | 354 |  | 8,074 | 392 |  | 5,256 | 549 |  | 5,994 | 478 |
| **P** | 1,546 | 54 |  | 1,889 | 49 |  | 2,087 | 35 |  | 1,939 | 32 |  | 2,148 | 62.80 |  | 1,978 | 41 |
| **Mn** | 568 | 23 |  | 606.4 | 44 |  | 491 | 47 |  | 510 | 27 |  | 423 | 36 |  | 622 | 118 |
| **Sn** | 17 | 4 |  | 20 | 4 |  | 17 | 4 |  | 19 | 4 |  | 17 | 3 |  | 21 | 4 |
| **Si** | 131,372 | 2,683 |  | 133,943 | 6,581 |  | 154,082 | 11,973 |  | 147,046 | 5,479 |  | 111,247 | 7,740 |  | 159,298 | 12,248 |
| **S** | 5,790 | 133 |  | 3,355 | 470 |  | 3,039 | 210 |  | 3,396 | 245 |  | 4,478 | 392 |  | 1,622 | 287 |
| **Ti** | 6,090 | 465 |  | 6,934 | 660 |  | 6,044 | 523 |  | 4,509 | 686.4 |  | 6,050 | 769 |  | 6,491 | 711 |
| **Rb** | 27 | 1 |  | 29 | 2 |  | 27.2 | 0.8 |  | 32.4 | 0.9 |  | 20 | 3 |  | 24 | 3 |
| **Sr** | 216 | 5 |  | 134 | 5 |  | 125 | 6 |  | 133 | 5 |  | 165 | 77 |  | 158 | 61 |
| **Y** | 12 | 1 |  | 14 | 2 |  | 11 | 2 |  | 11 | 1 |  | 8 | 2 |  | 11 | 1 |
| **Nb** | 2.2 | 0.4 |  | 4.2 | 0.8 |  | 3.6 | 0.5 |  | 2.6 | 0.5 |  | < 2 |  |  | 5 | 1 |
| **Mo** | 5 | 1 |  | 10 | 1 |  | 4.4 | 0.9 |  | 9.6 | 0.9 |  | 7 | 2 |  | 7 | 3 |
| **Ba** | 244 | 6 |  | 294 | 25 |  | 244 | 14 |  | 331 | 32 |  | 291 | 17 |  | 285 | 16 |

**Table S3.** EPA 3050B extraction results for the two size fractions studied at each site. Data reported in ppm, as determined by ICP-OES. Values < LOQ are marked with “-”.

| **Site** | **Fraction^1^** | **Al** | **V** | **Cr** | **Fe** | **Ni** | **Cu** | **Zn** | **Pb** | **As** | **Cd** |
| --- | --- | --- | --- | --- | --- | --- | --- | --- | --- | --- | --- |
| **1** | **C** | 4,800 | 42 | 14 | 8,100 | 7.5 | 51 | 93 | 9.2 | 0.5 | 1.3 |
| **4** | **C** | 3,600 | 34 | 44 | 14,000 | 15 | 230 | 270 | 40 | 0.6 | 2.2 |
| **5** | **C** | 3,000 | 15 | 15 | 8,800 | 6.3 | 79 | 150 | 17 | 0.2 | 1.3 |
| **6** | **C** | 3,000 | 14 | 52 | 18,000 | 14 | 120 | 390 | 68 | - | 2.4 |
| **12** | **C** | 2,800 | 19 | 37 | 10,000 | 15 | 130 | 260 | 28 | 0.8 | 1.5 |
| **21** | **C** | 2,400 | 14 | 73 | 15,000 | 13 | 92 | 180 | 27 | 0.1 | 1.9 |
|  |  |  |  |  |  |  |  |  |  |  |  |
| **1** | **F** | 6,700 | 61 | 16 | 10,000 | 8.4 | 90 | 150 | 19 | 0.3 | 1.6 |
| **4** | **F** | 5,000 | 46 | 66 | 17,000 | 23 | 200 | 460 | 59 | 0.4 | 2.8 |
| **5** | **F** | 5,700 | 34 | 44 | 13,000 | 18 | 210 | 520 | 67 | - | 2.4 |
| **6** | **F** | 5,300 | 20 | 51 | 15,000 | 18 | 180 | 690 | 140 | 1.5 | 2.3 |
| **12** | **F** | 4,100 | 31 | 66 | 13,000 | 25 | 190 | 570 | 72 | 1.3 | 2.0 |
| **21** | **F** | 4,600 | 25 | 60 | 14,000 | 29 | 180 | 560 | 82 | 0.8 | 2.2 |

^1^ C: coarse (<841 µm); F: fine (<75 µm).

**Table S4**. Comparison of descriptive statistics of XRF results for selected elements in six bulk road dusts, which were studied by O’Shea et al. (2020) and in the present investigation. Both datasets are for material from the same sampling locations. All values listed as ppm.

|  | **O'Shea et al. (2020)** | | | | | **This study** | | | | |
| --- | --- | --- | --- | --- | --- | --- | --- | --- | --- | --- |
| **Element** | **Min.** | **Median** | **Max.** | **Mean** | **SD^1^** | **Min.** | **Median** | **Max.** | **Mean** | **SD^1^** |
| **Al** | 18,000 | 20,000 | 29,000 | 22,000 | 4,400 | 20,000 | 24,000 | 27,000 | 24,000 | 2,600 |
| **Fe** | 40,000 | 54,000 | 104,000 | 65,000 | 28,000 | 34,000 | 41,000 | 48,000 | 41,000 | 6,100 |
| **V** | 90 | 190 | 320 | 190 | 84 | <1 | 120 | 251 | 130 | 81 |
| **Cr** | 51 | 140 | 270 | 160 | 87 | 53 | 180 | 270 | 170 | 75 |
| **Cu** | 130 | 310 | 1,200 | 460 | 420 | 130 | 180 | 250 | 190 | 42 |
| **Zn** | 200 | 660 | 2,900 | 1,000 | 1,000 | 310 | 950 | 1,300 | 830 | 350 |
| **Pb** | 34 | 200 | 690 | 290 | 240 | 21 | 71 | 120 | 69 | 35 |

^1^SD = Standard Deviation

**Table S5.** Percentage of element concentrations in the EPA 3050B extract (% Extr.) relative to their bulk concentration in the respective road-dust samples. The reported standard deviation (SD) reflects concentration variabilities in the bulk road dust only, and therefore, represents a minimum estimate of the total uncertainty. EPA 3050 data for size fraction <841 µm.

| **Element** | **Site 1** | |  | **Site 4** | |  | **Site 5** | |  | **Site 6** | |  | **Site 12** | |  | **Site 21** | |
| --- | --- | --- | --- | --- | --- | --- | --- | --- | --- | --- | --- | --- | --- | --- | --- | --- | --- |
|  | % Extr. | SD |  | % Extr. | SD |  | % Extr. | SD |  | % Extr. | SD |  | % Extr. | SD |  | % Extr. | SD |
| **Al** | 18.8 | 0.6 |  | 15.6 | 0.7 |  | 13.3 | 0.3 |  | 11.7 | 0.4 |  | 14 | 4 |  | 9 | 2 |
| **Fe** | 21.9 | 0.5 |  | 29 | 2 |  | 26 | 2 |  | 39 | 2 |  | 29 | 1 |  | 34 | 2 |
| **V** | 17 | 2 |  | 21 | 4 |  | 12 | 2 |  |  |  |  | 17 | 2 |  | 12 | 2 |
| **Cr** | 26 | 4 |  | 21 | 3 |  | 11 | 4 |  | 28 | 8 |  | 22 | 6 |  | 27 | 2 |
| **Ni** | 24 | 2 |  | 34 | 10 |  | 21 | 4 |  | 41 | 4 |  | 34 | 8 |  | 30 | 9 |
| **Cu** | 40 | 12 |  | 94 | 32 |  | 48 | 31 |  | 55 | 16 |  | 67 | 44 |  | 53 | 23 |
| **Zn** | 30 | 2 |  | 27 | 5 |  | 28 | 5 |  | 30 | 4 |  | 28 | 5 |  | 19 | 6 |
| **Pb** | 44 | 4 |  | 64 | 11 |  | 36 | 11 |  | 55 | 9 |  | 35 | 8 |  | 32 | 6 |


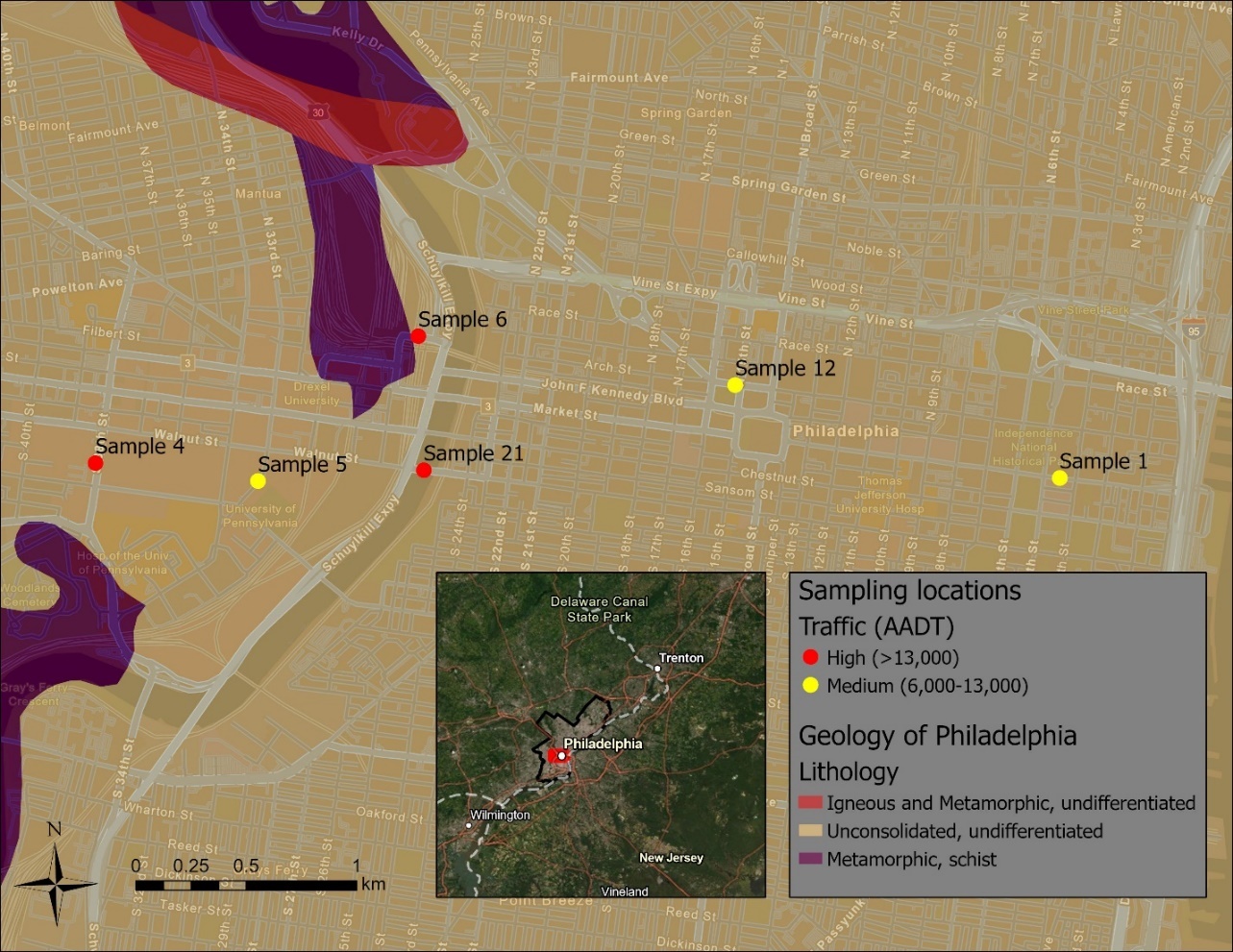


Igneous & Metamorphic, undifferentiated

Unconsolidated, undifferentiated

Metamorphic, Wissahickon schist

**Figure S1.** Locations of the road dust sampling sites in Philadelphia. The sample (or “sites” referred in the text) names are identical to those used in O’Shea et al. (2020), as the material was collected at the same sites. Lithologic characteristics are based on Berg et al. (1980). AADT = average annual daily traffic.


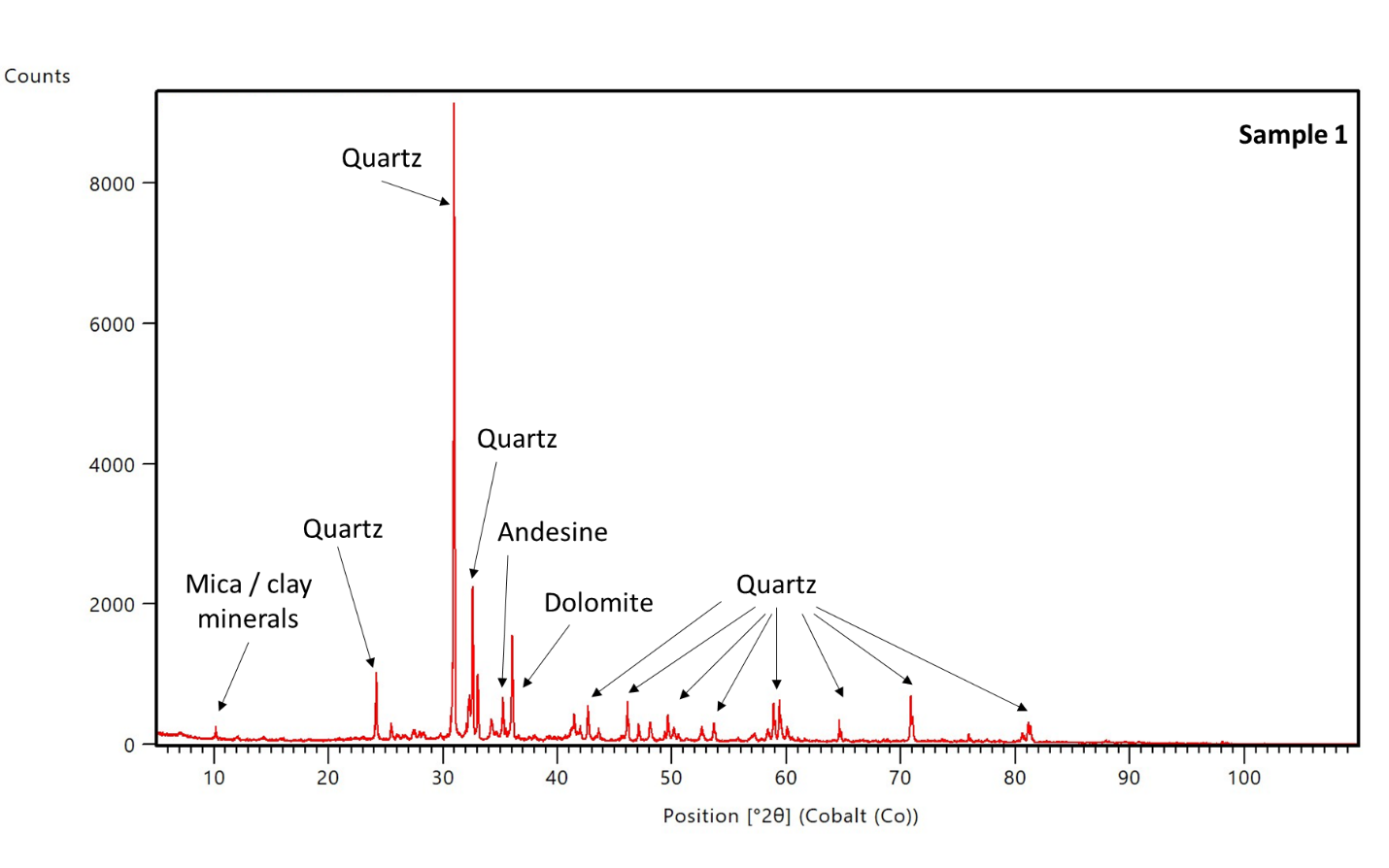
**Figure S2.** Site 1: XRD pattern and mineralogical interpretation.

**Site 1**


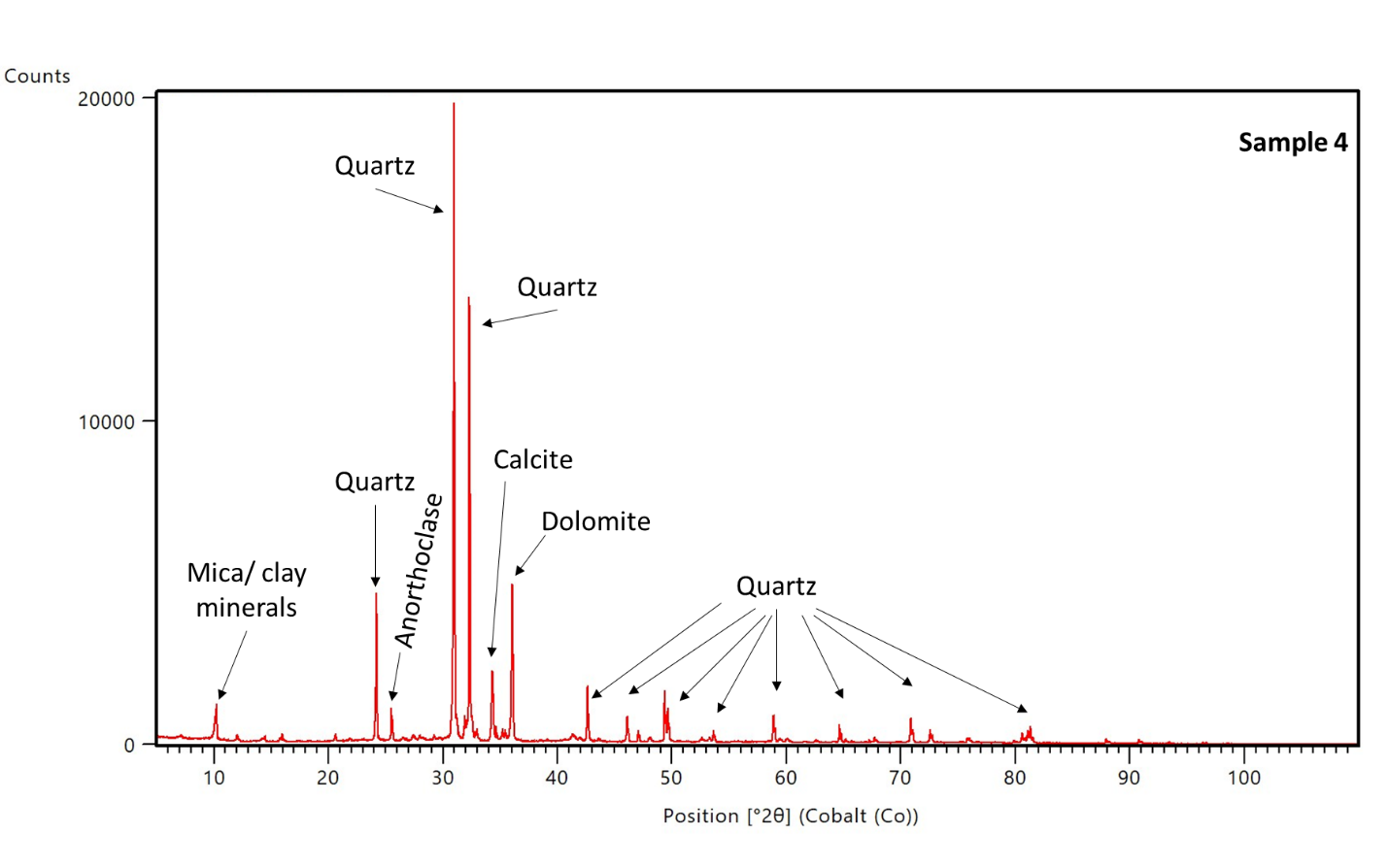
**Figure S3.** Site 4: XRD pattern and mineralogical interpretation.

**Site 4**


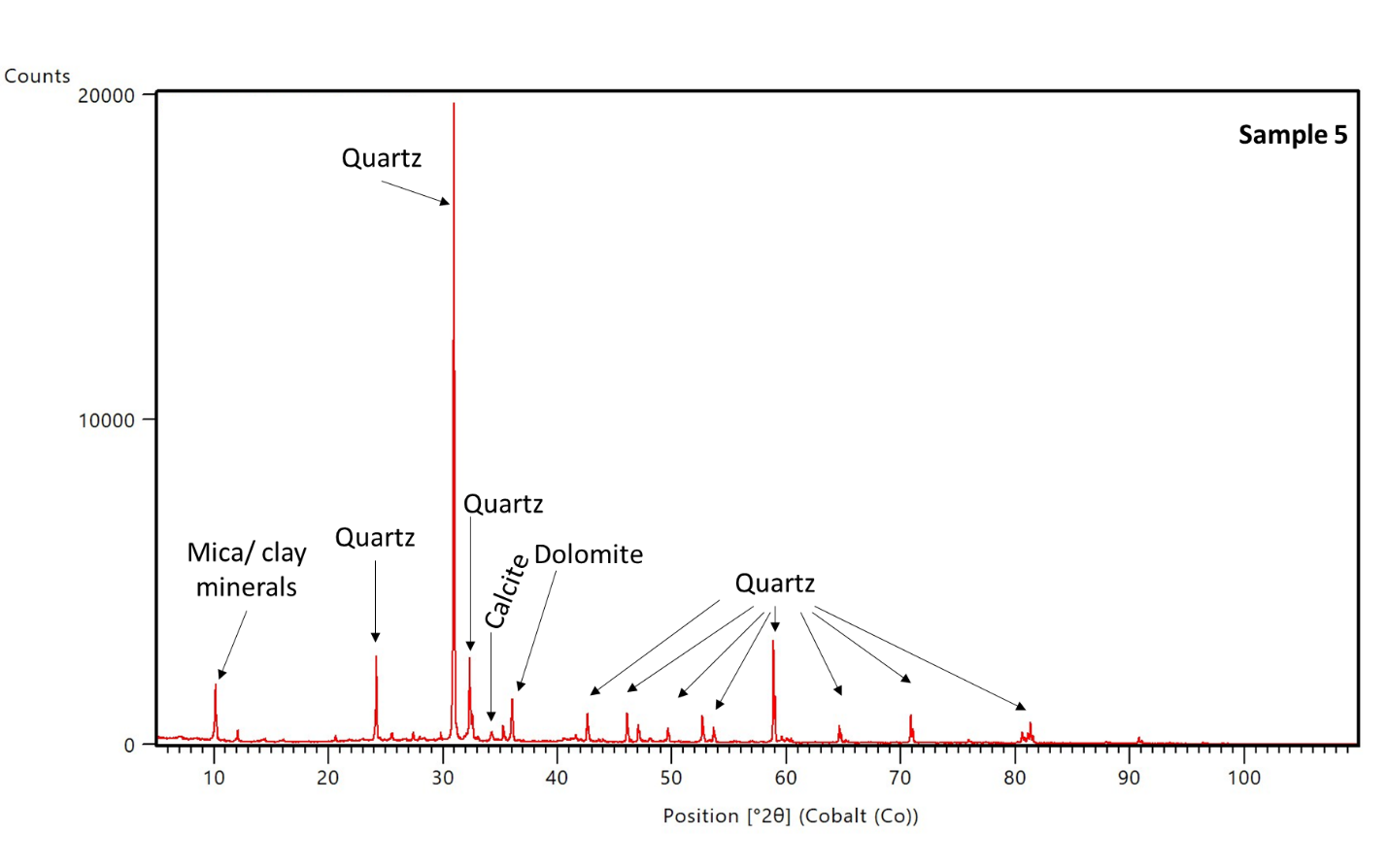
**Figure S4.** Site 5: XRD pattern and mineralogical interpretation.

**Site 5**


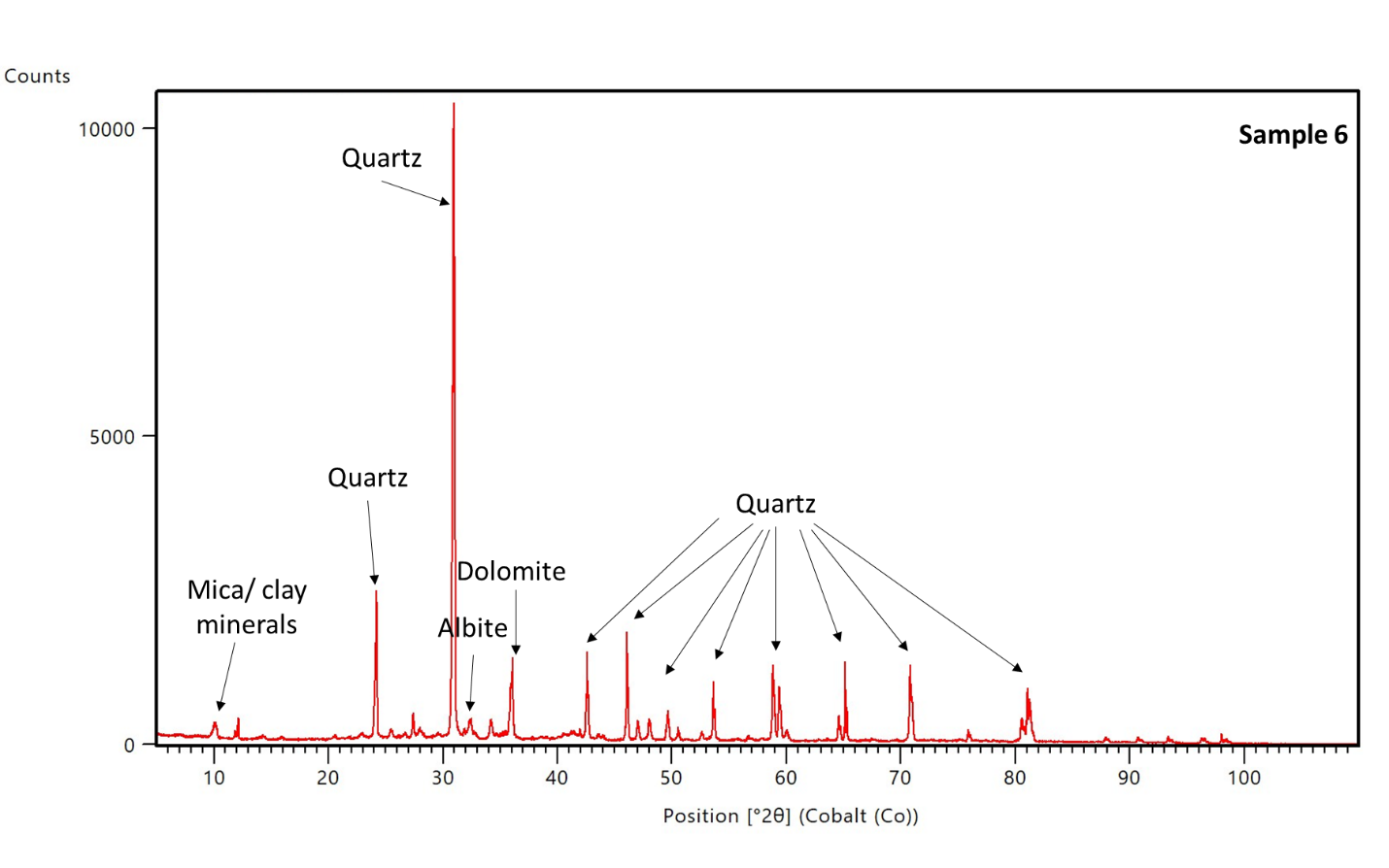
**Figure S5.** Site 6: XRD pattern and mineralogical interpretation.

**Site 6**


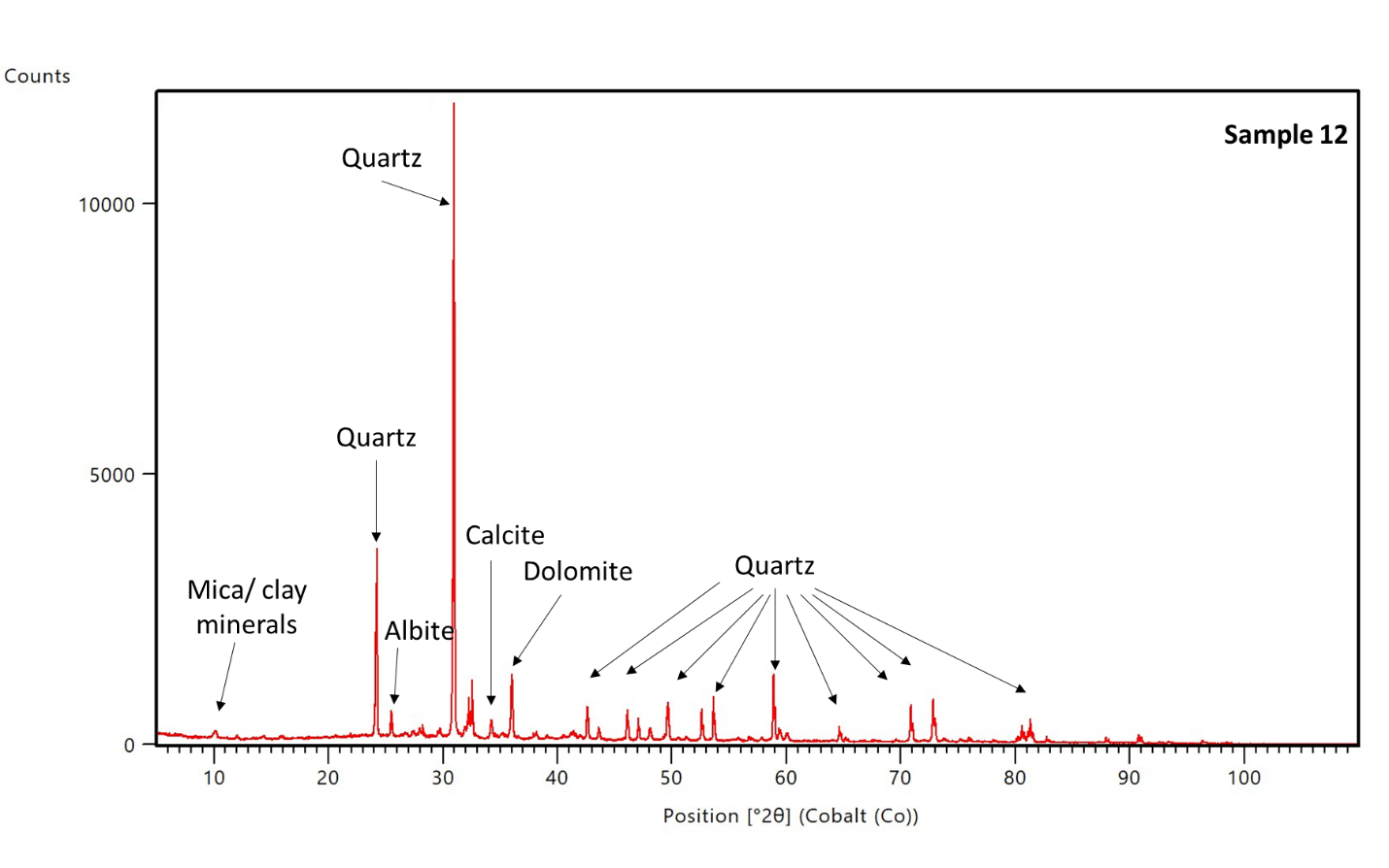
**Figure S6.** Site 12: XRD pattern and mineralogical interpretation.

**Site 12**


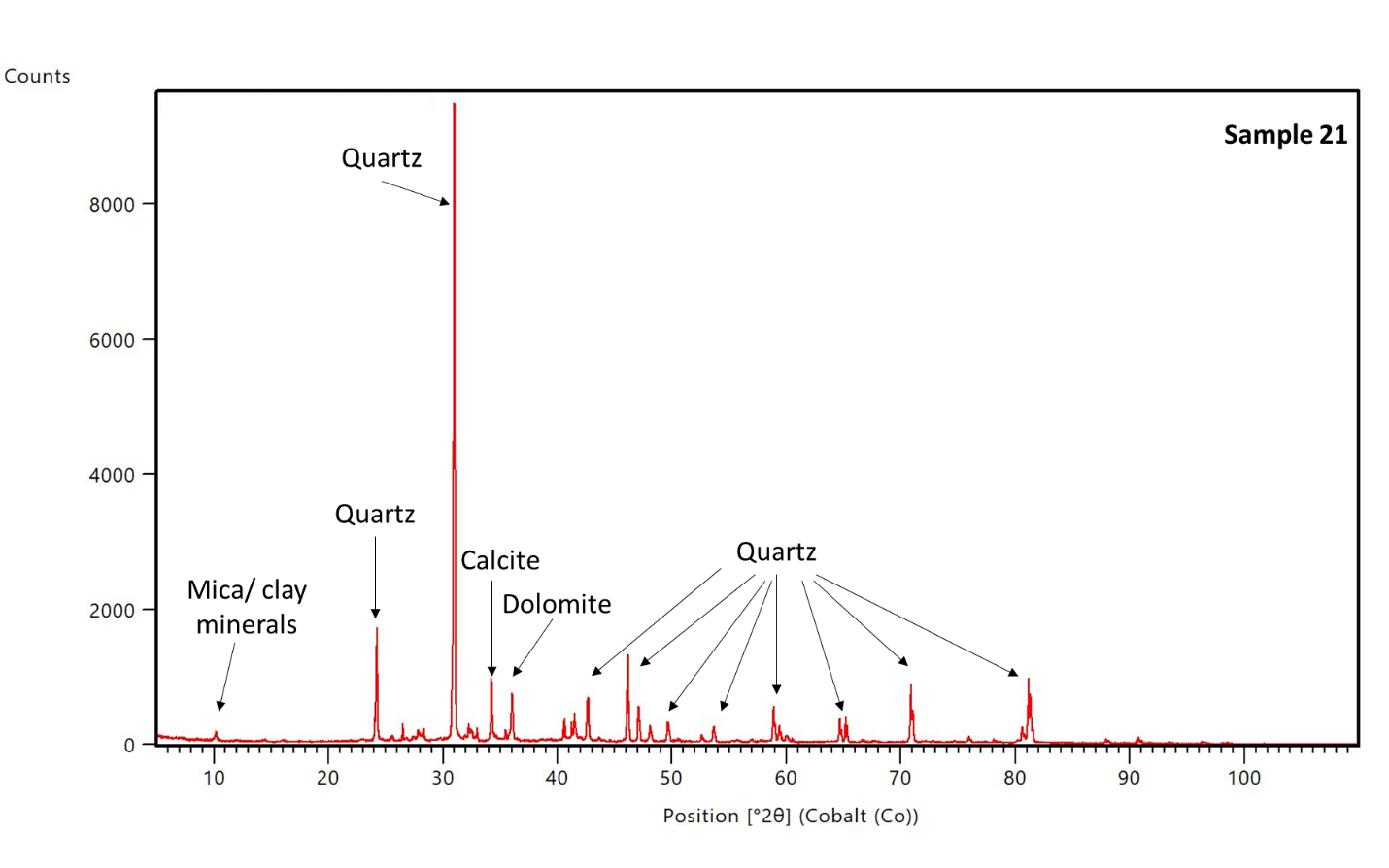
**Figure S7.** Site 21: XRD pattern and mineralogical interpretation.

**Site 21**


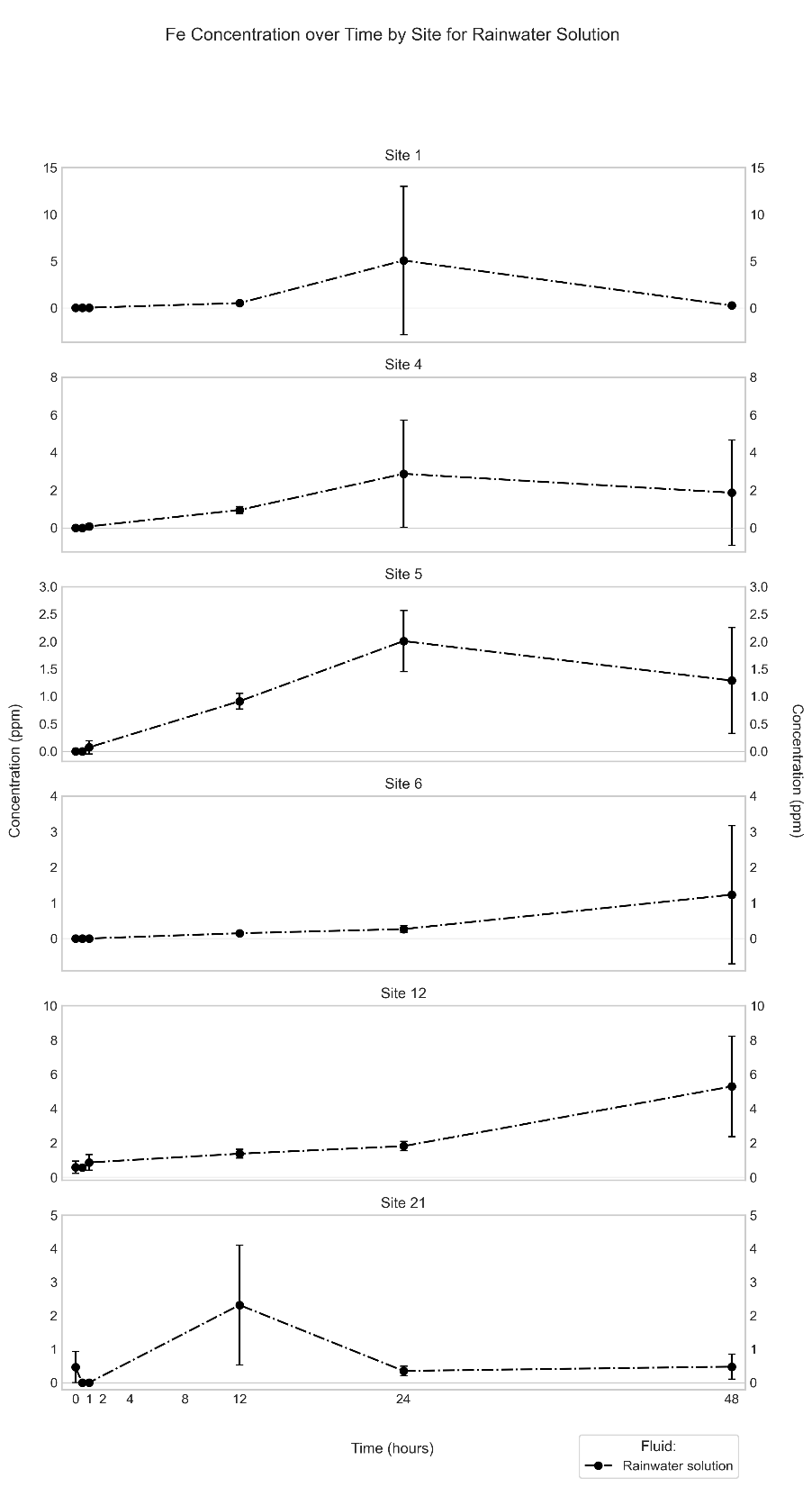


**Figure S8.** Mean (*n* = 3) Fe concentration in synthetic rainwater extract (in ppm) through time for the different sites.


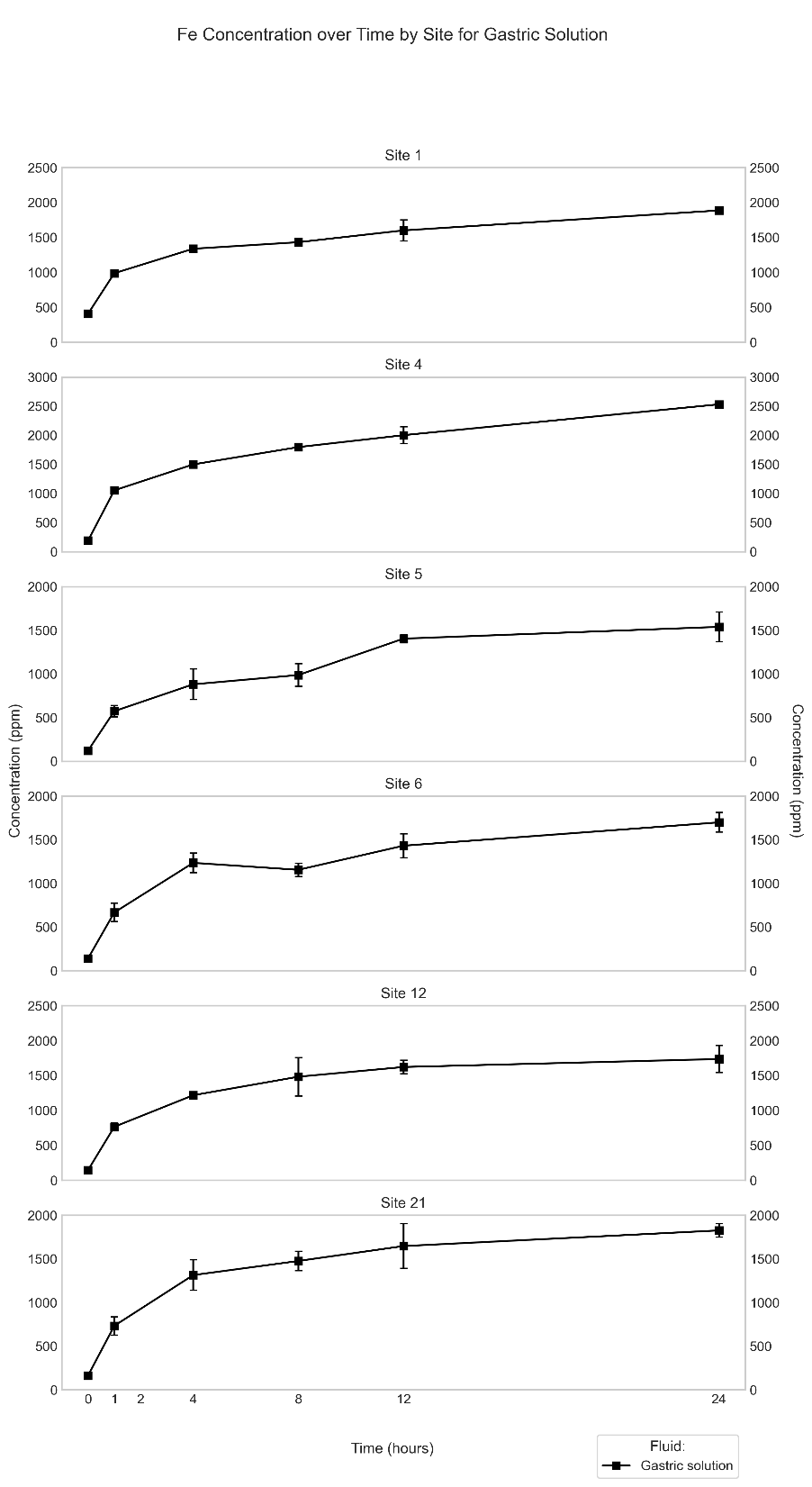


**Figure S9.** Mean (*n* = 3) Fe concentration in simulated gastric fluid extract (in ppm) through time for the different sites.


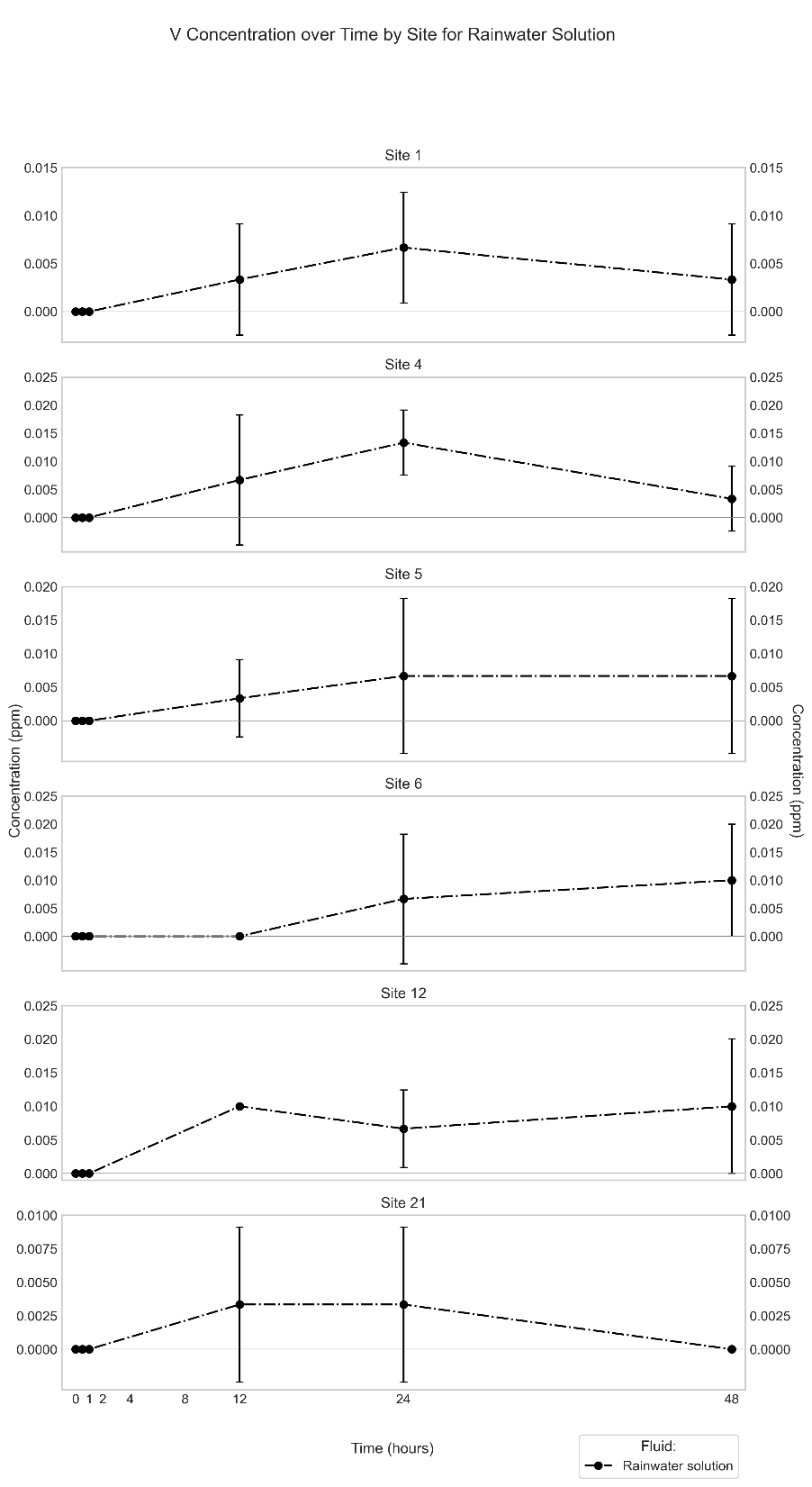


**Figure S10.** Mean (*n* = 3) V concentration in synthetic rainwater extract (in ppm) through time for the different sites.


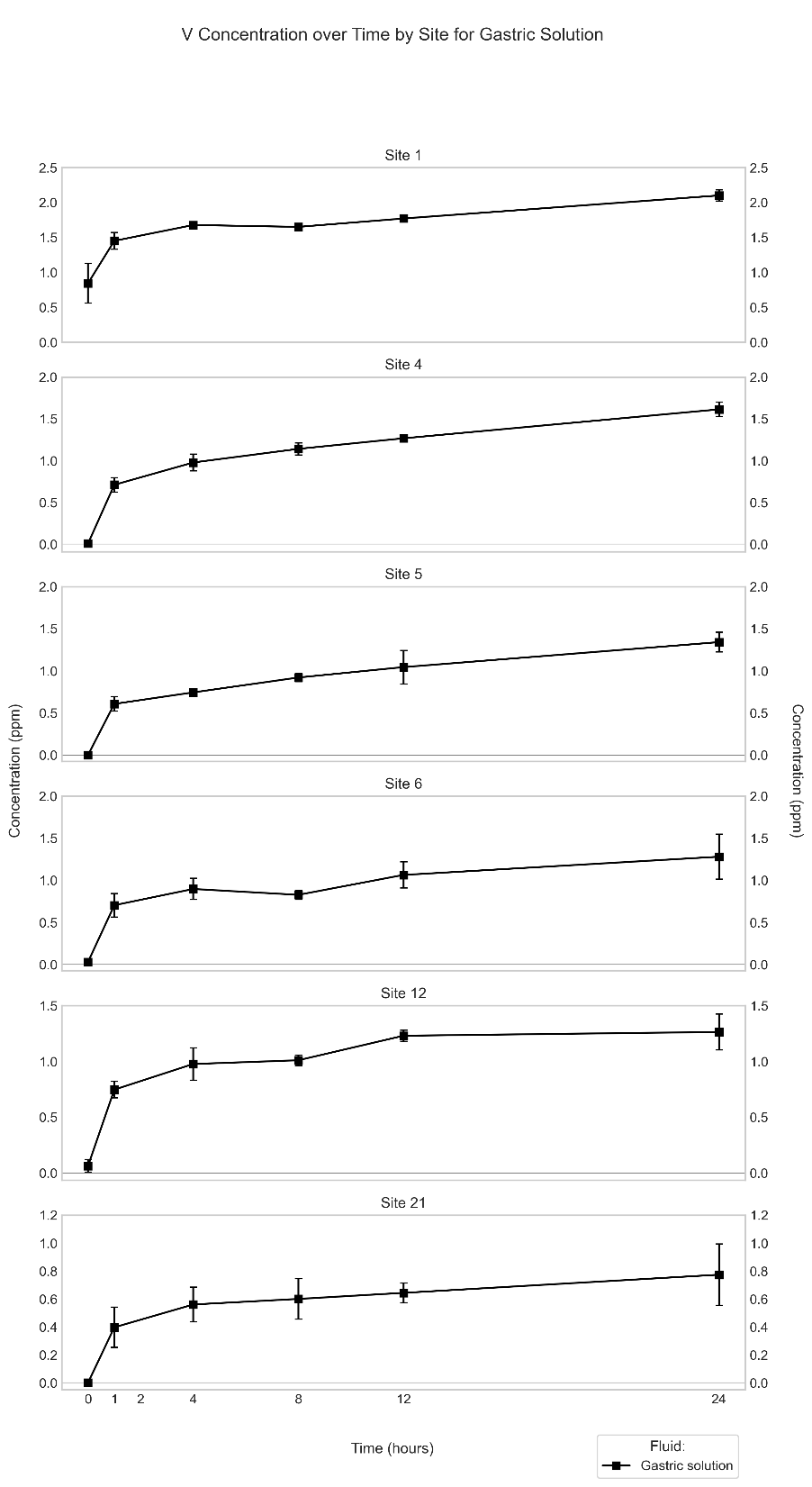


**Figure S11.** Mean (*n* = 3) V concentration in simulated gastric fluid extract (in ppm) through time for the different sites.


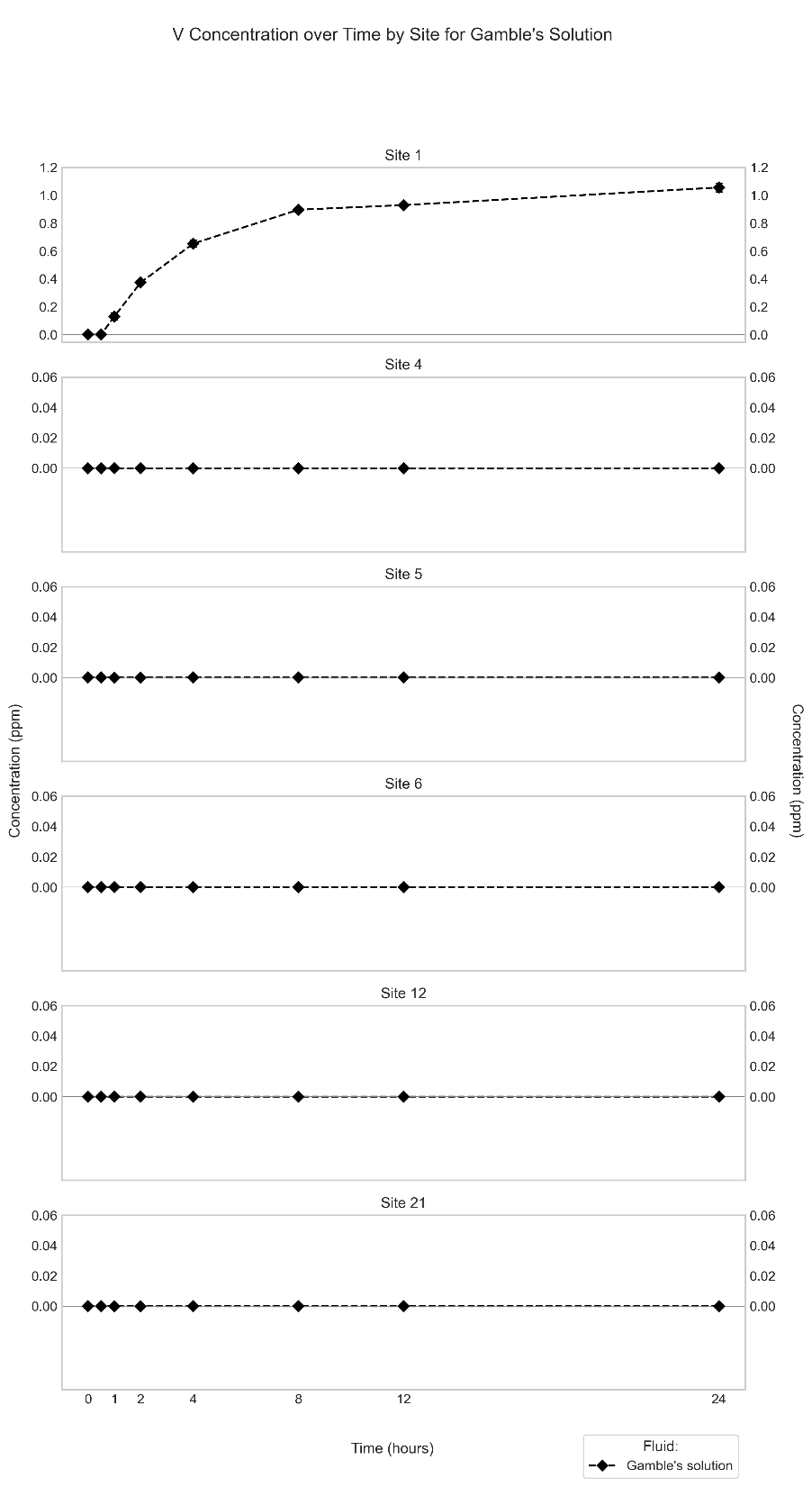


**Figure S12.** Mean (n = 3) V concentration in Gamble’s solution extract (in ppm) through time for the different sites. Note that quantifiable V was extracted only from Site-1 road dust.


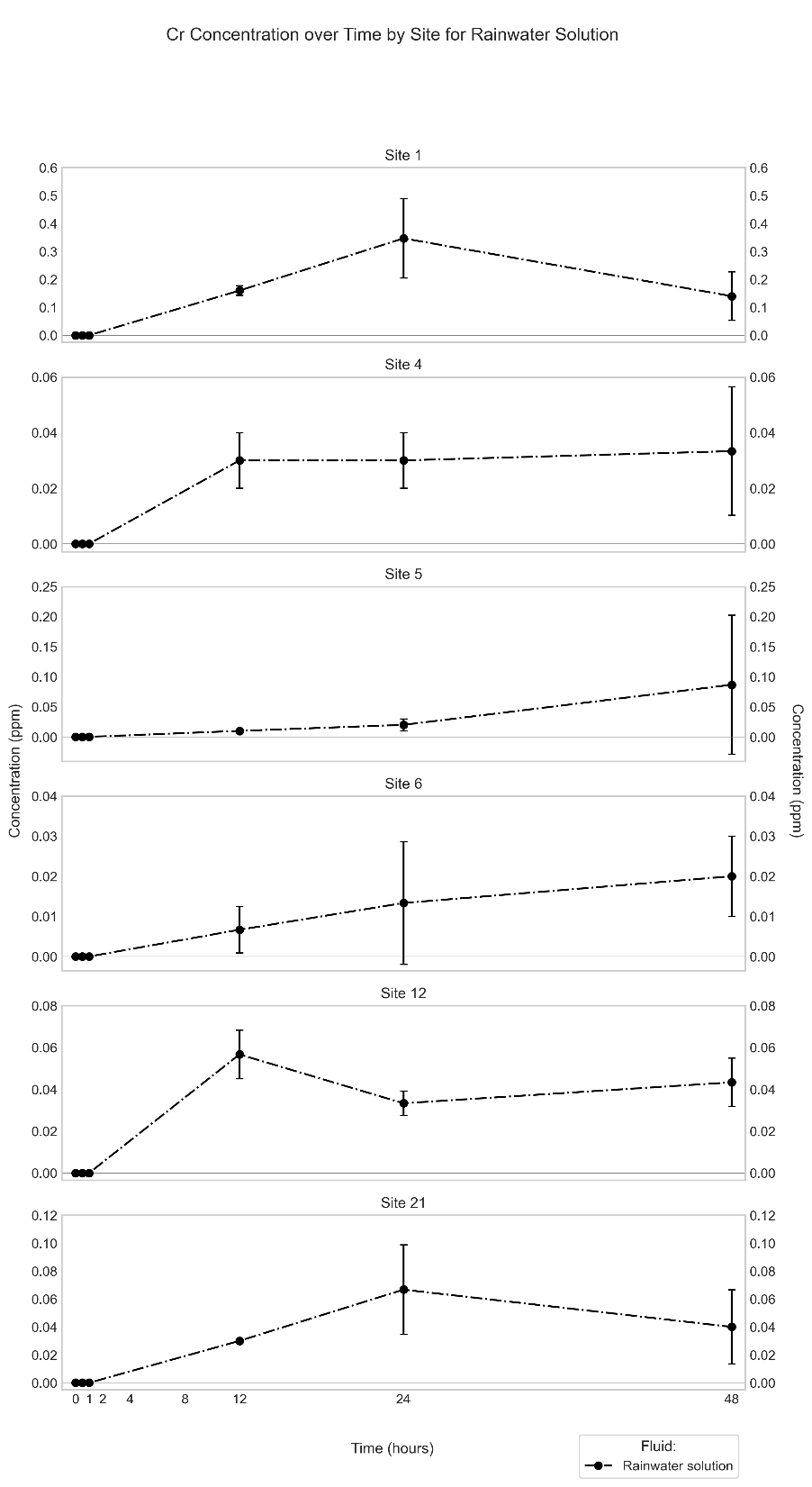


**Figure S13.** Mean (*n* = 3) Cr concentration in synthetic rainwater extract (in ppm) through time for the different sites.


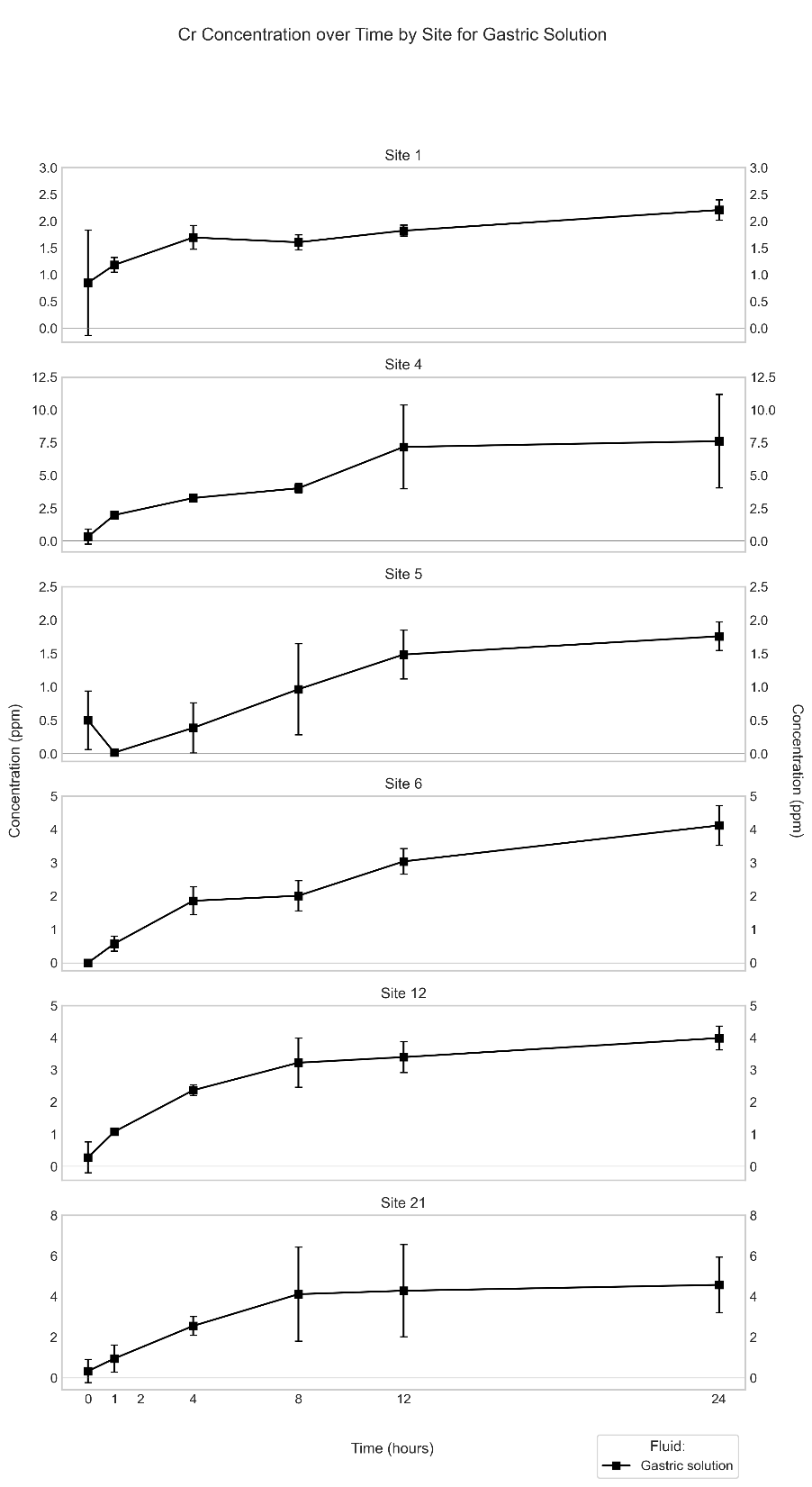


**Figure S14.** Mean (*n* = 3) Cr concentration in simulated gastric fluid extract (in ppm) through time for the different sites.


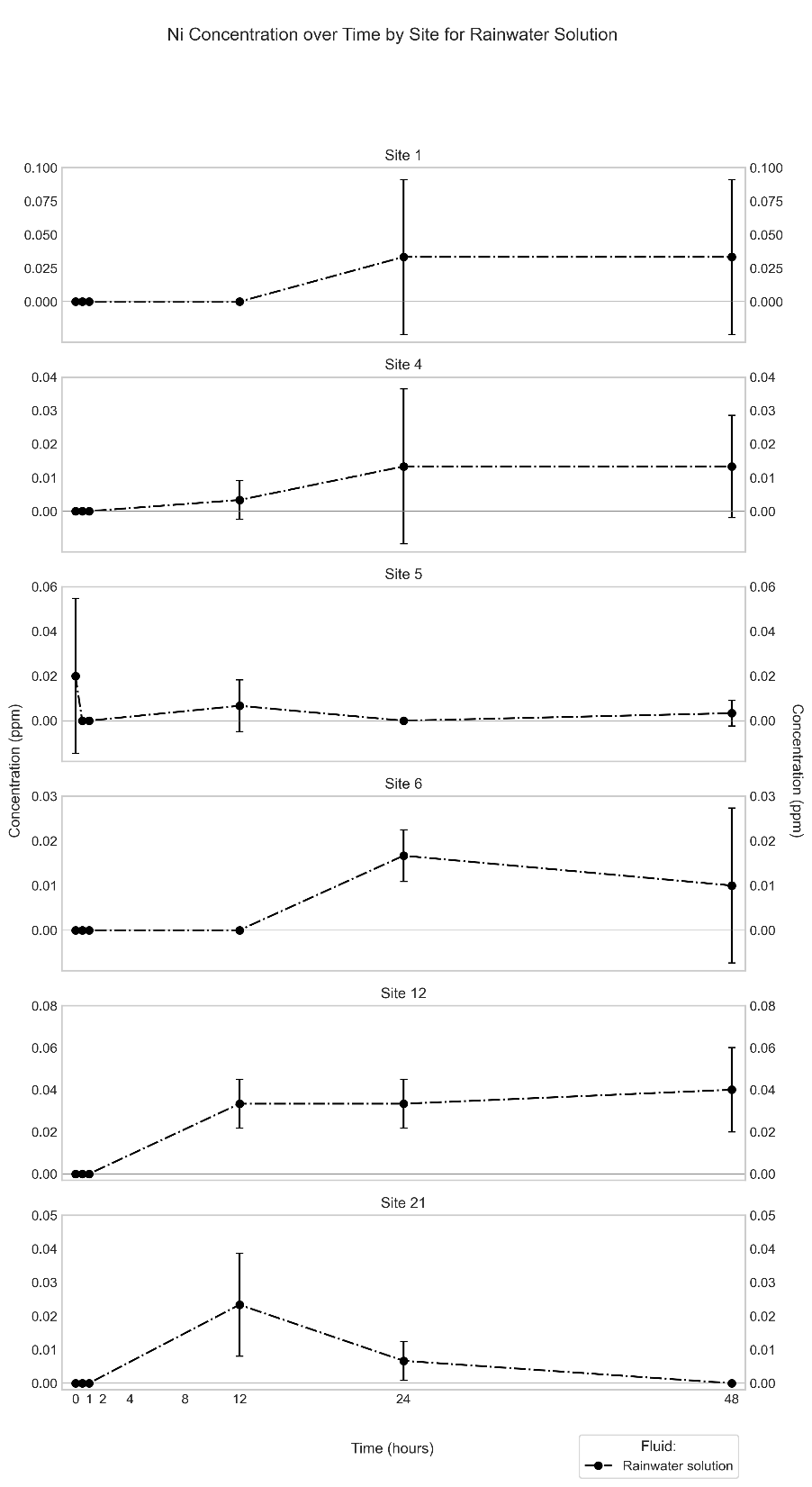


**Figure S15.** Mean (*n* = 3) Ni concentration in synthetic rainwater extract (in ppm) through time for the different sites.


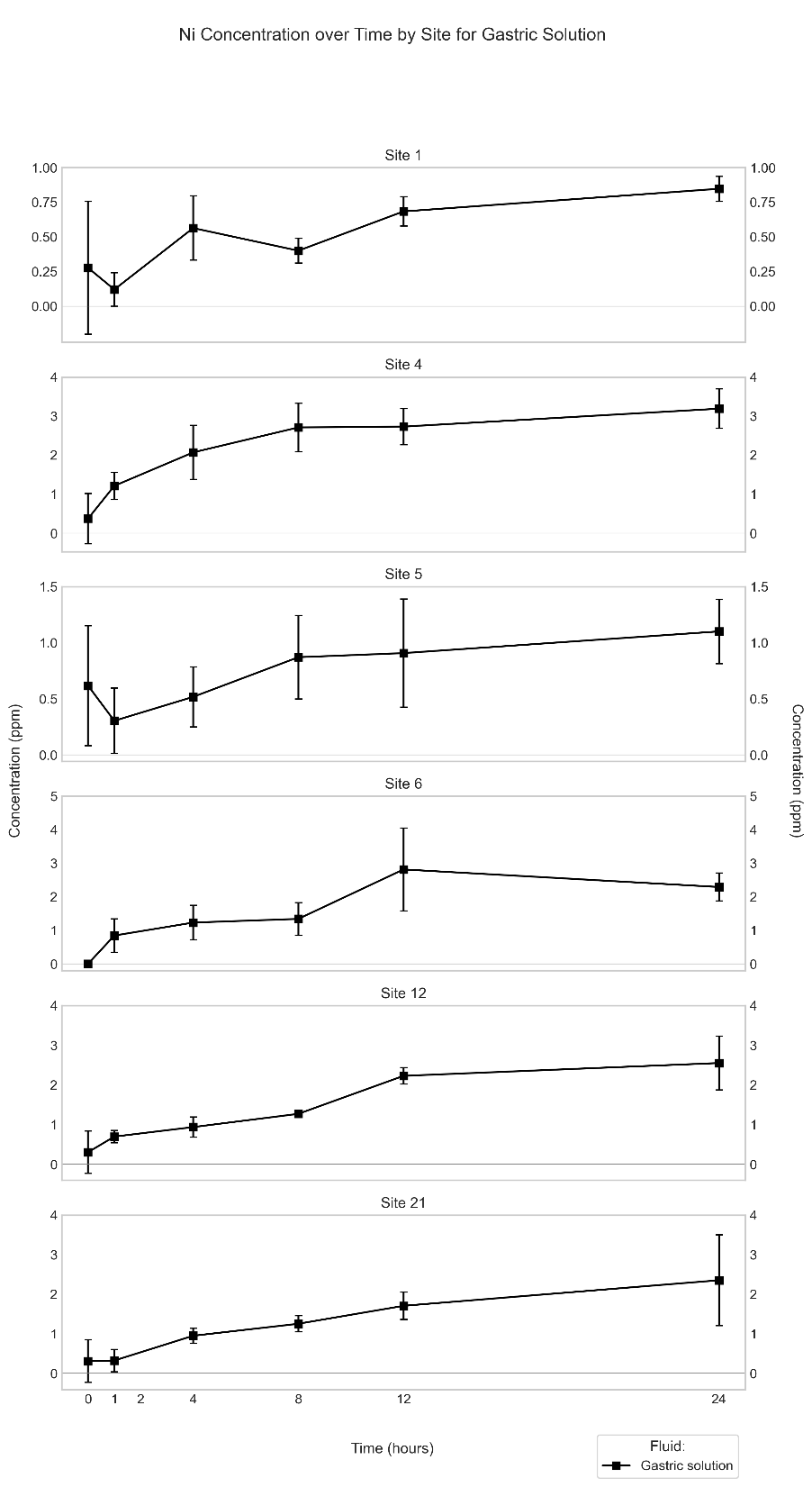


**Figure S16.** Mean (*n* = 3) Ni concentration in simulated gastric fluid extract (in ppm) through time for the different sites.


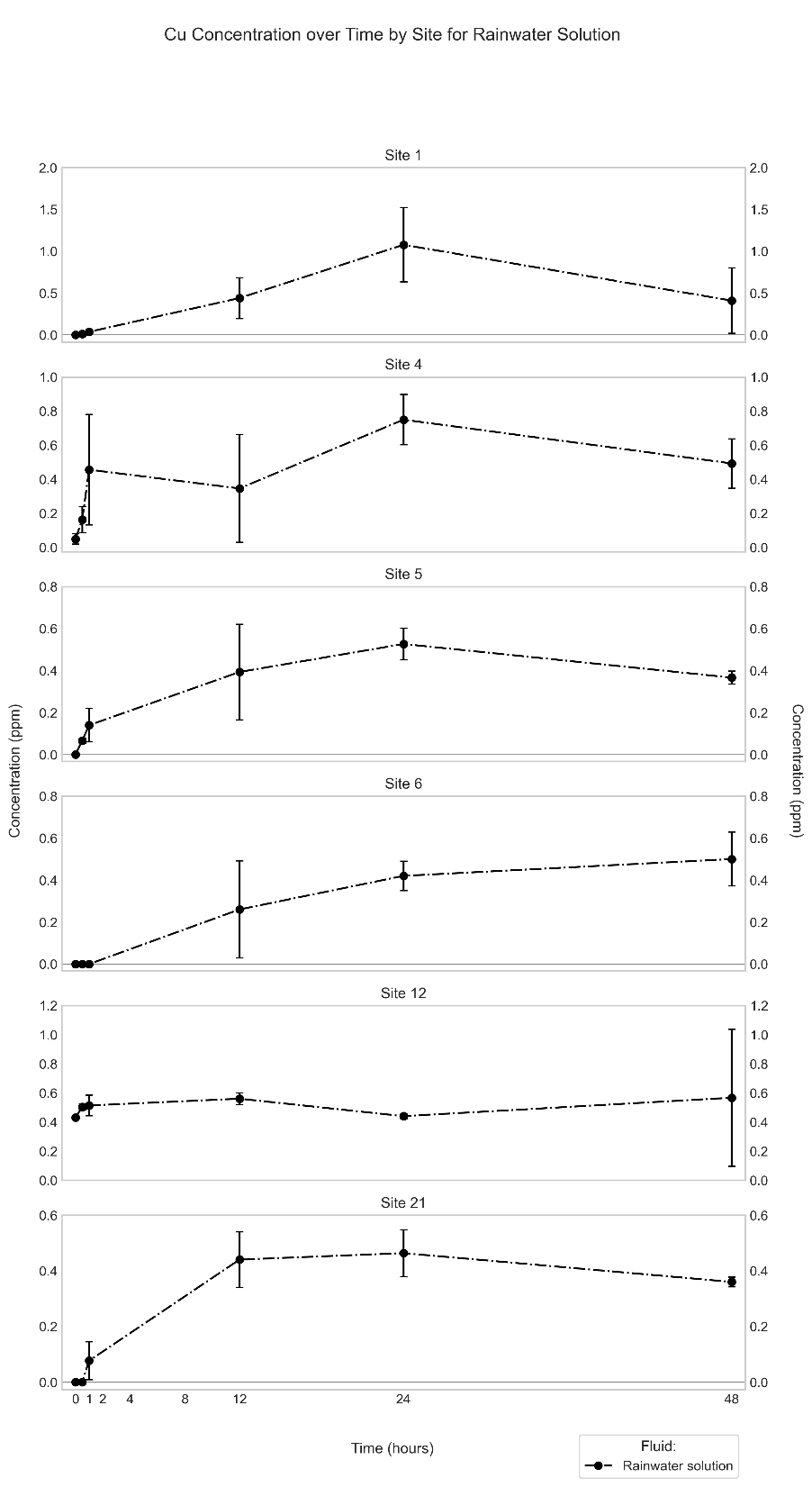


**Figure S17.** Mean (*n* = 3) Cu concentration in synthetic rainwater extract (in ppm) through time for the different sites.


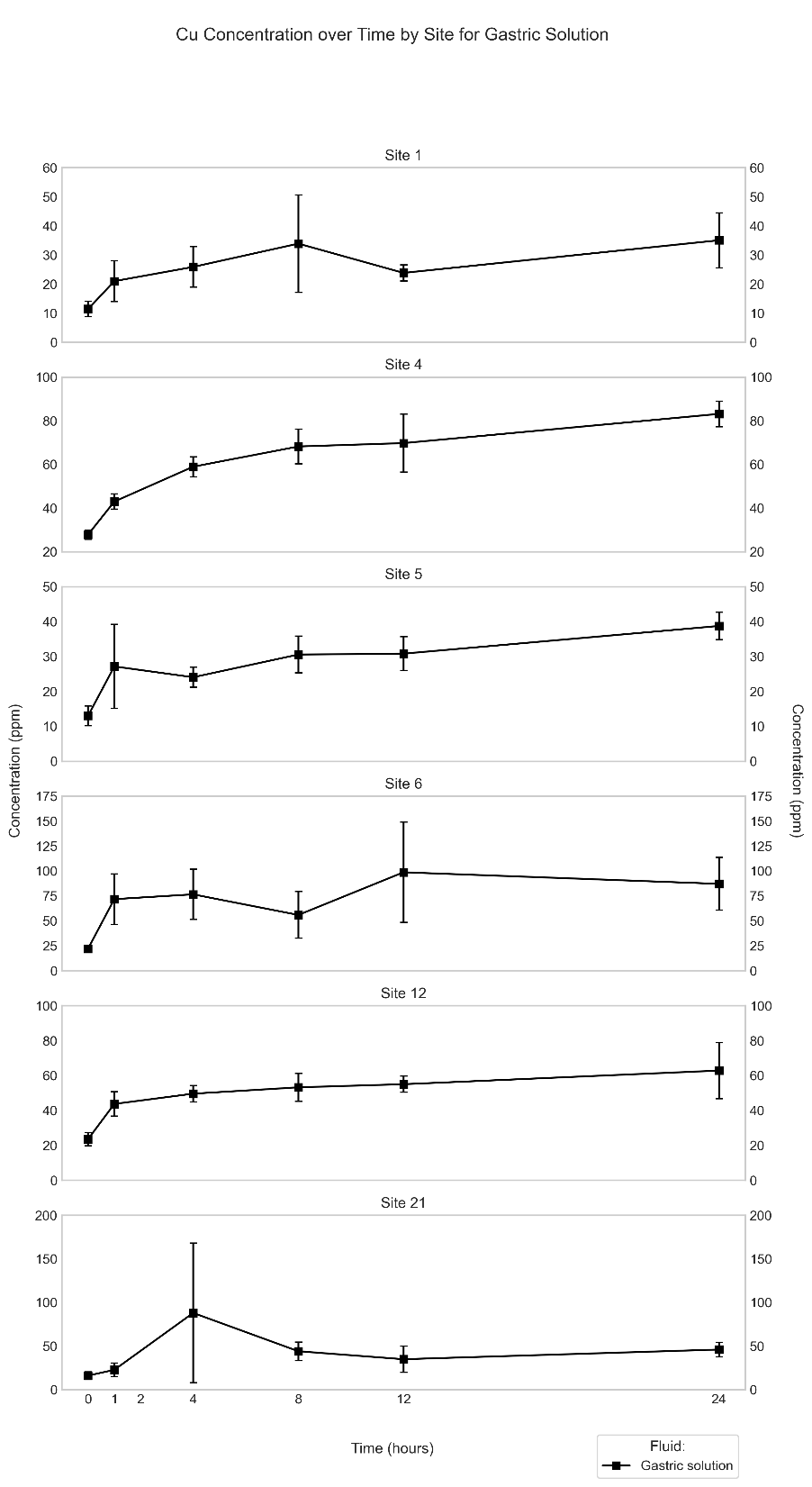


**Figure S18.** Mean (*n* = 3) Cu concentration in simulated gastric fluid extract (in ppm) through time for the different sites.


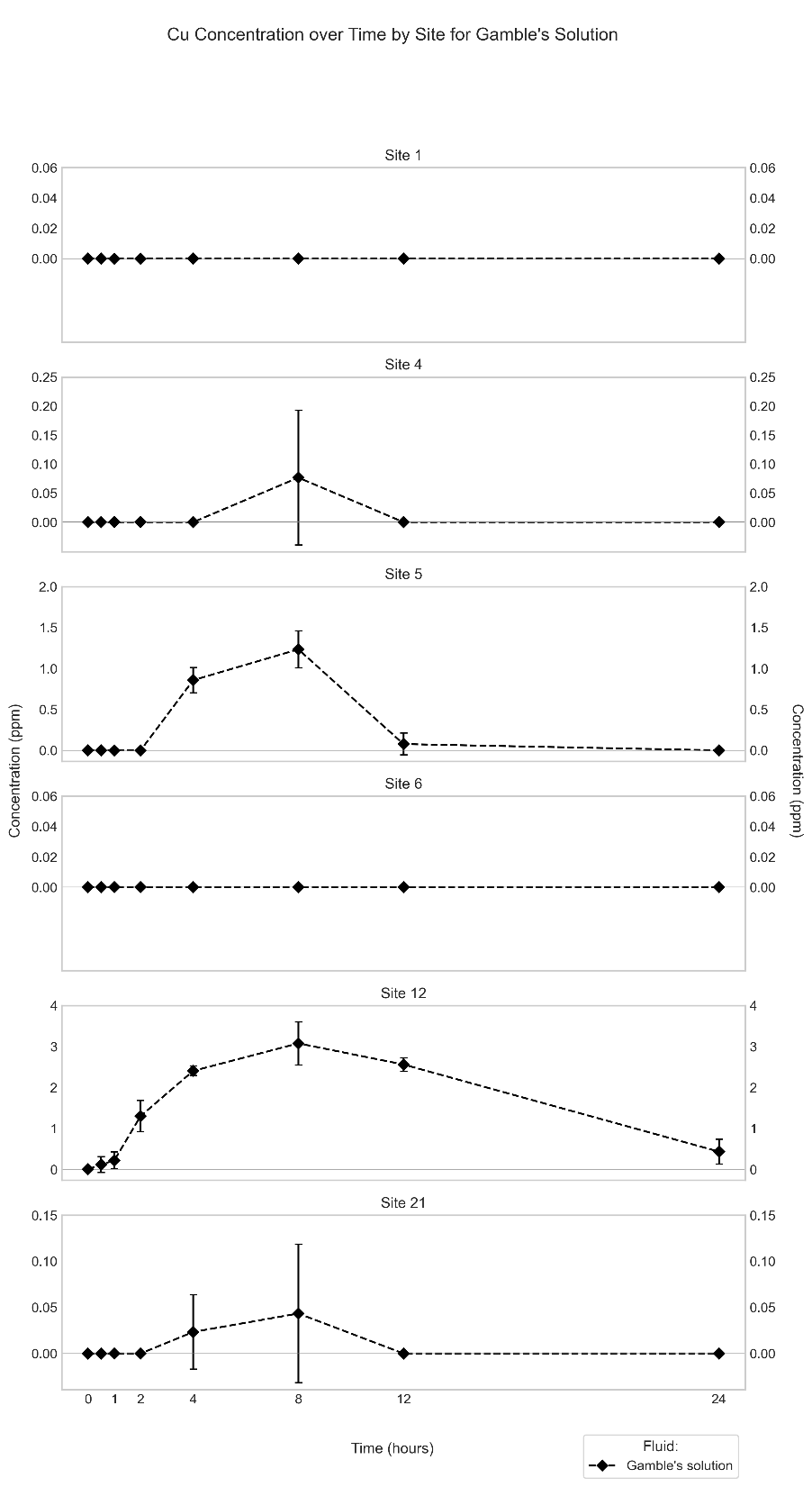


**Figure S19.** Mean (*n* = 3) Cu concentration in Gamble’s solution extract (in ppm) through time for the different sites.


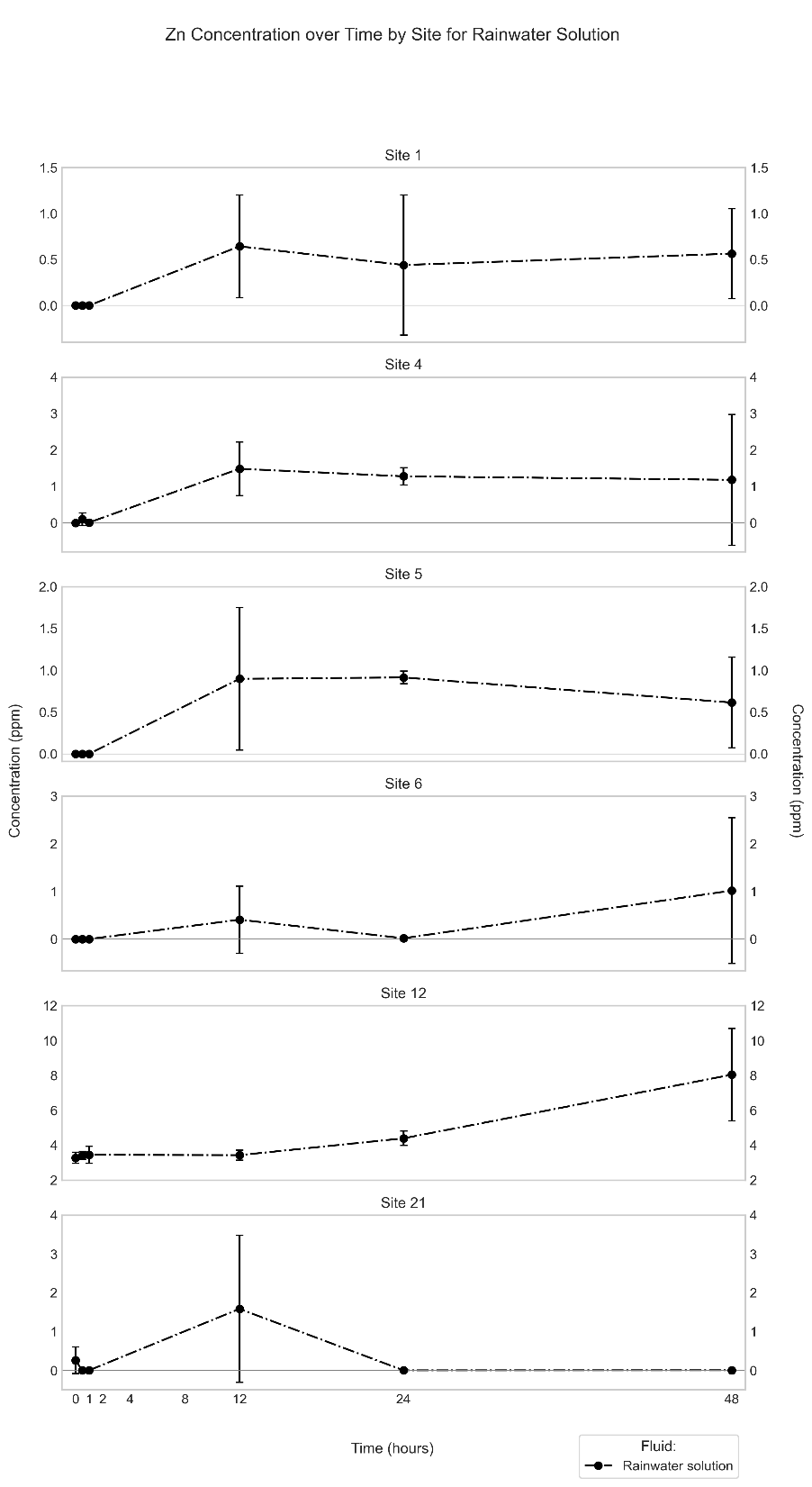


**Figure S20.** Mean (n = 3) Zn concentration in synthetic rainwater extract (in ppm) through time for the different sites.


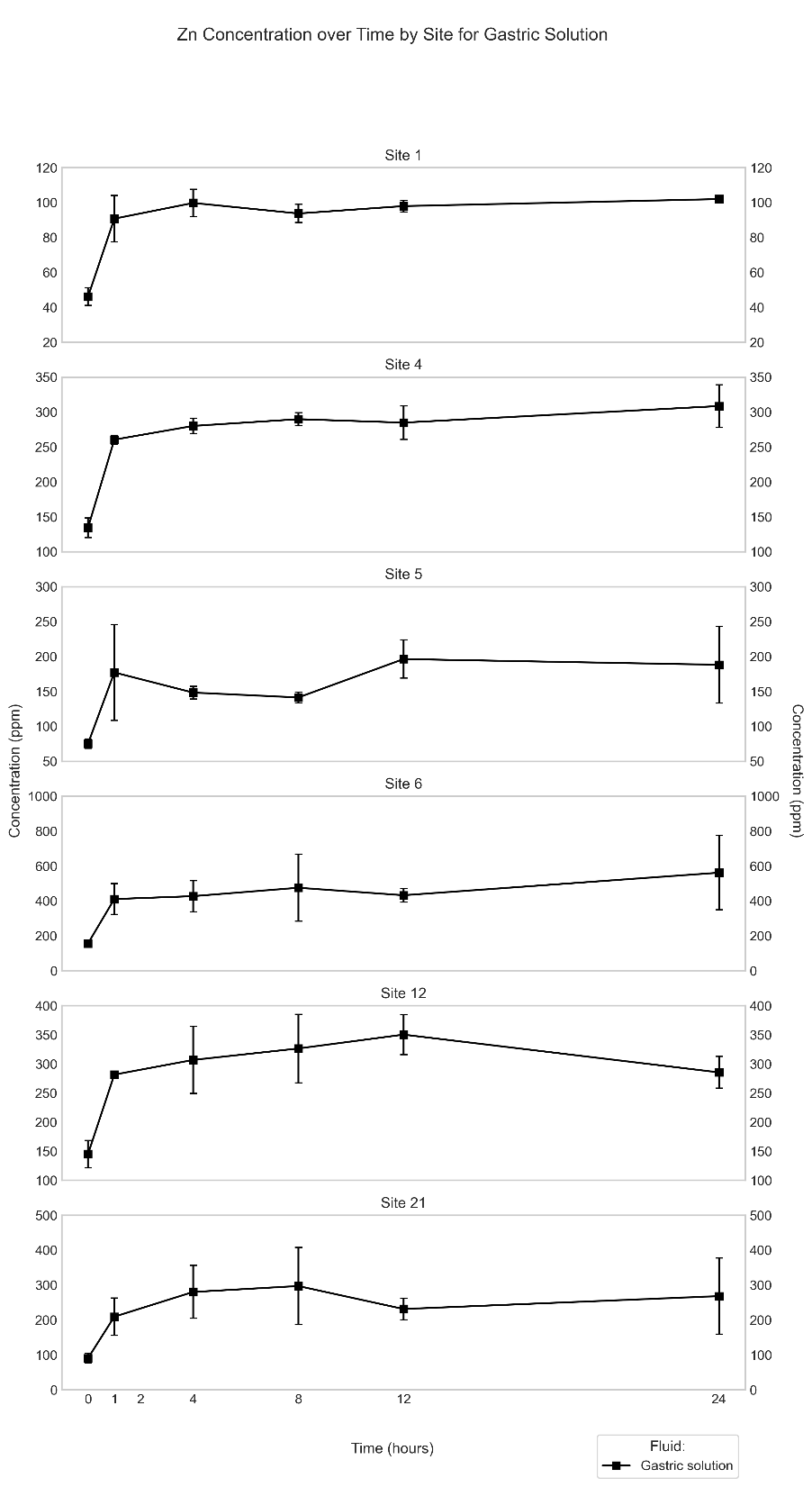


**Figure S21.** Mean (*n* = 3) Zn concentration in simulated gastric fluid experiment (in ppm) through time for the different sites.


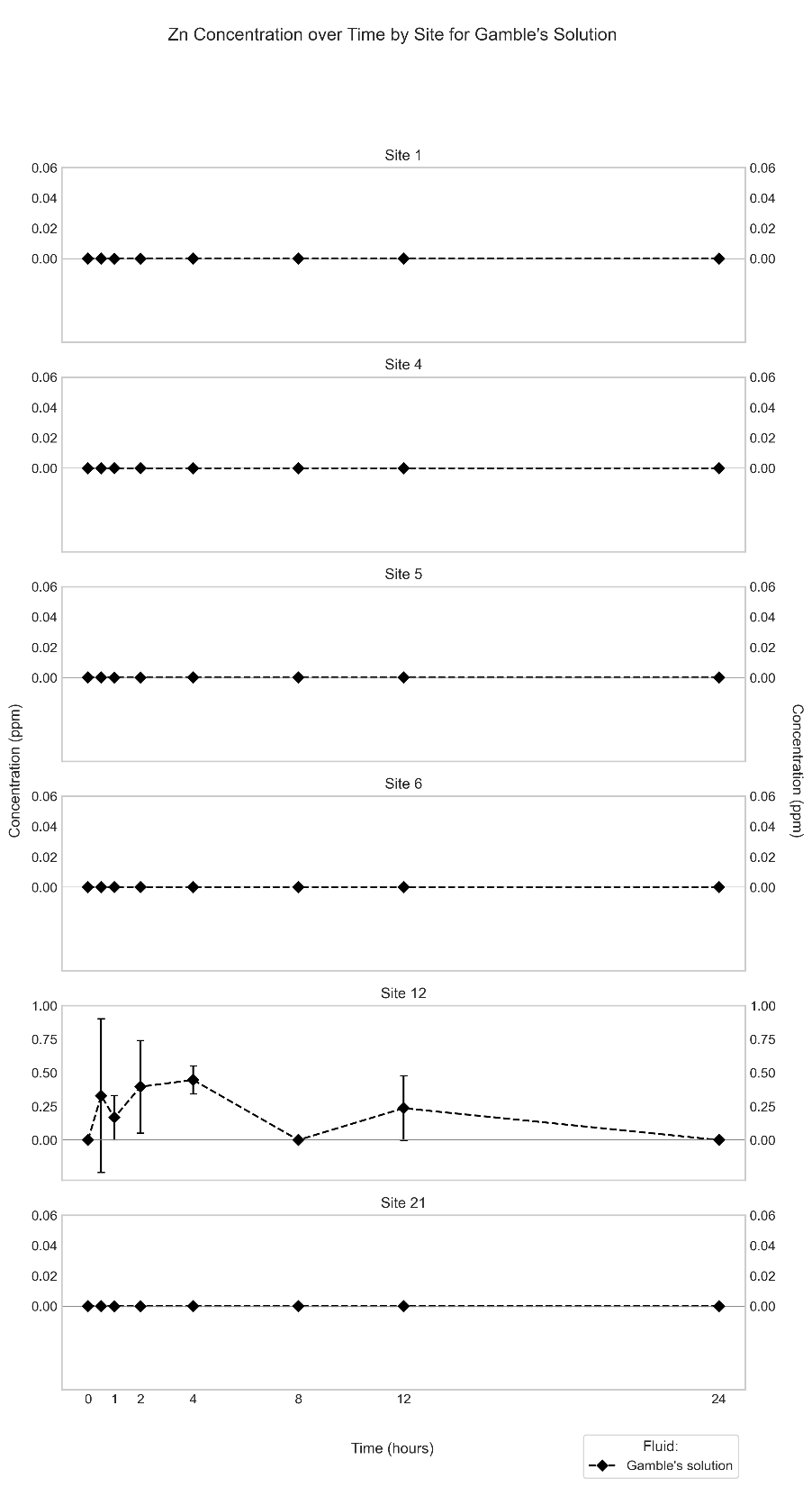


**Figure S22.** Mean (*n* = 3) Zn concentration in Gamble’s solution extract (in ppm) through time for the different sites. Note that quantifiable Zn was extracted only from Site-12 road dust.


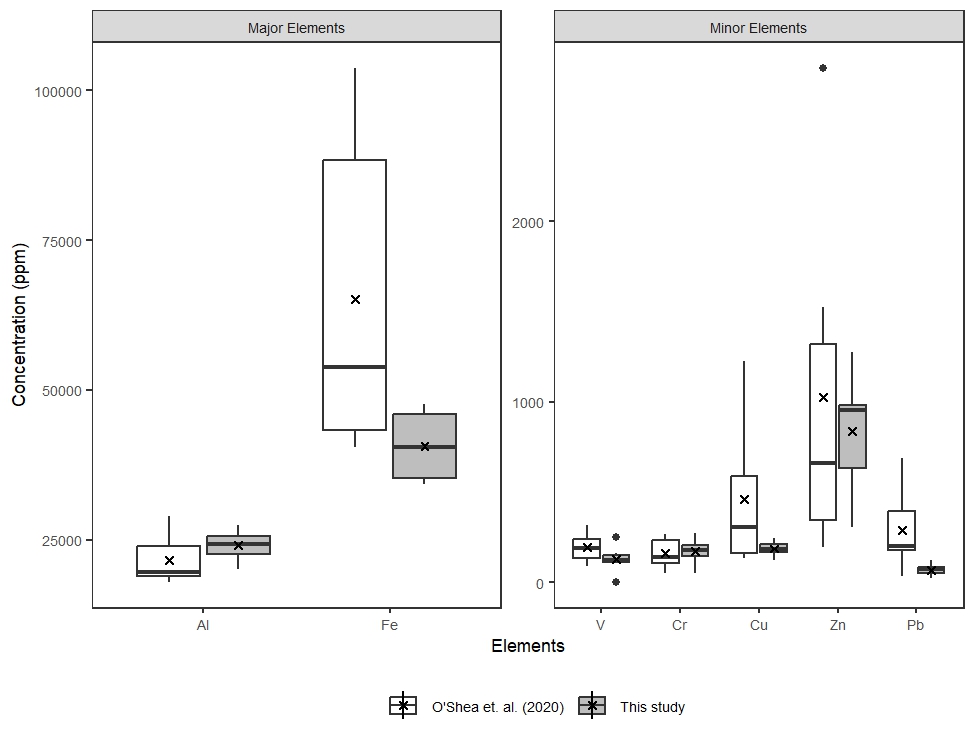


**Figure S23.** Comparison of element contents reported in both O’Shea et al. (2020) and this study for bulk road dust from the same six sampling locations. Cross (“x”) symbol inside the box refers to the mean concentration of each element from all six sites, horizontal line represents the median, and star symbols are outliers. Data are listed Table S4.


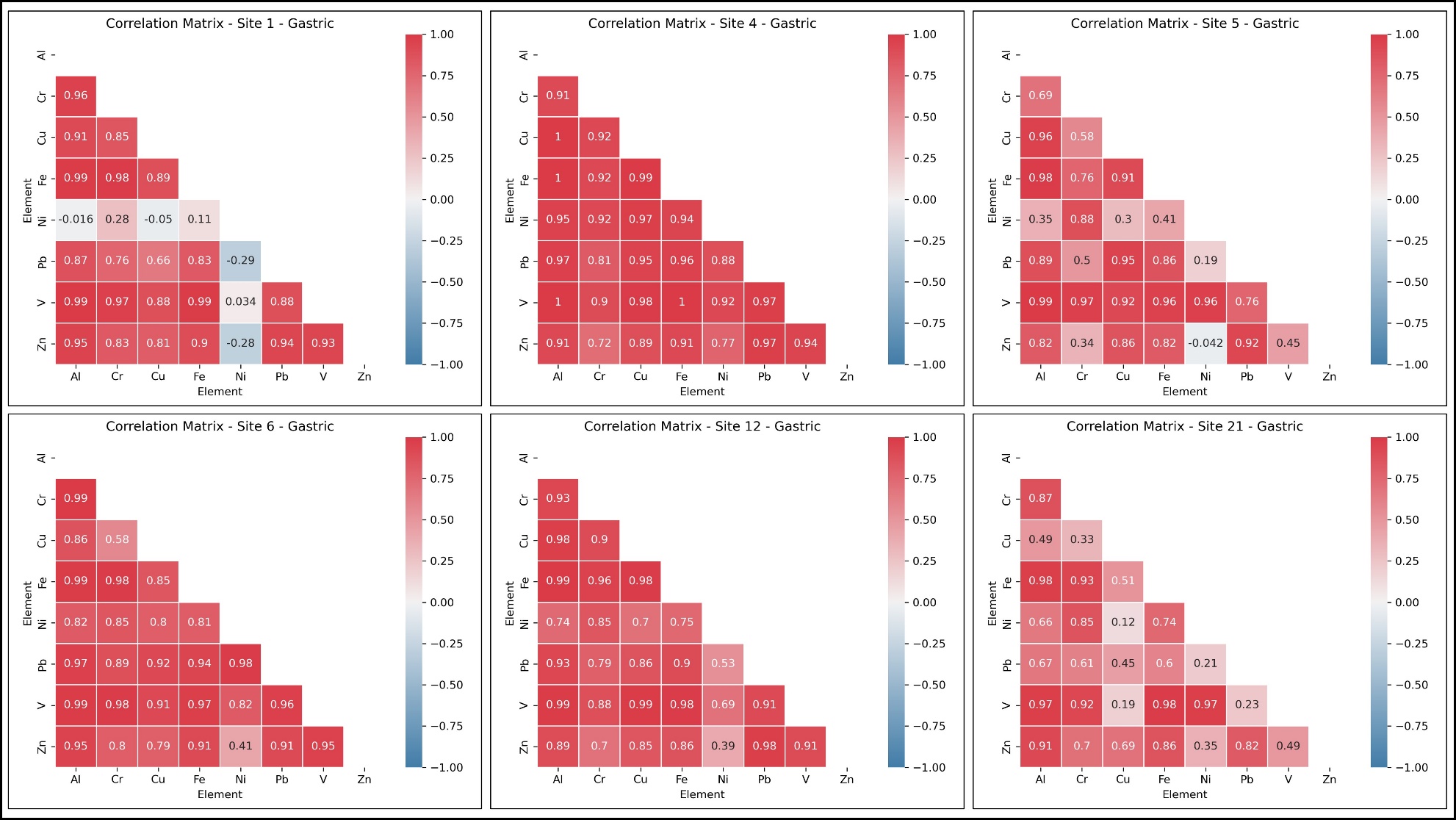


**Figure S24.** Pearson correlation matrices for the concentrations of the studied elements in the *simulated gastric-fluid* extracts from all six sites. Data points with element concentrations below their respective detection limits were not considered.


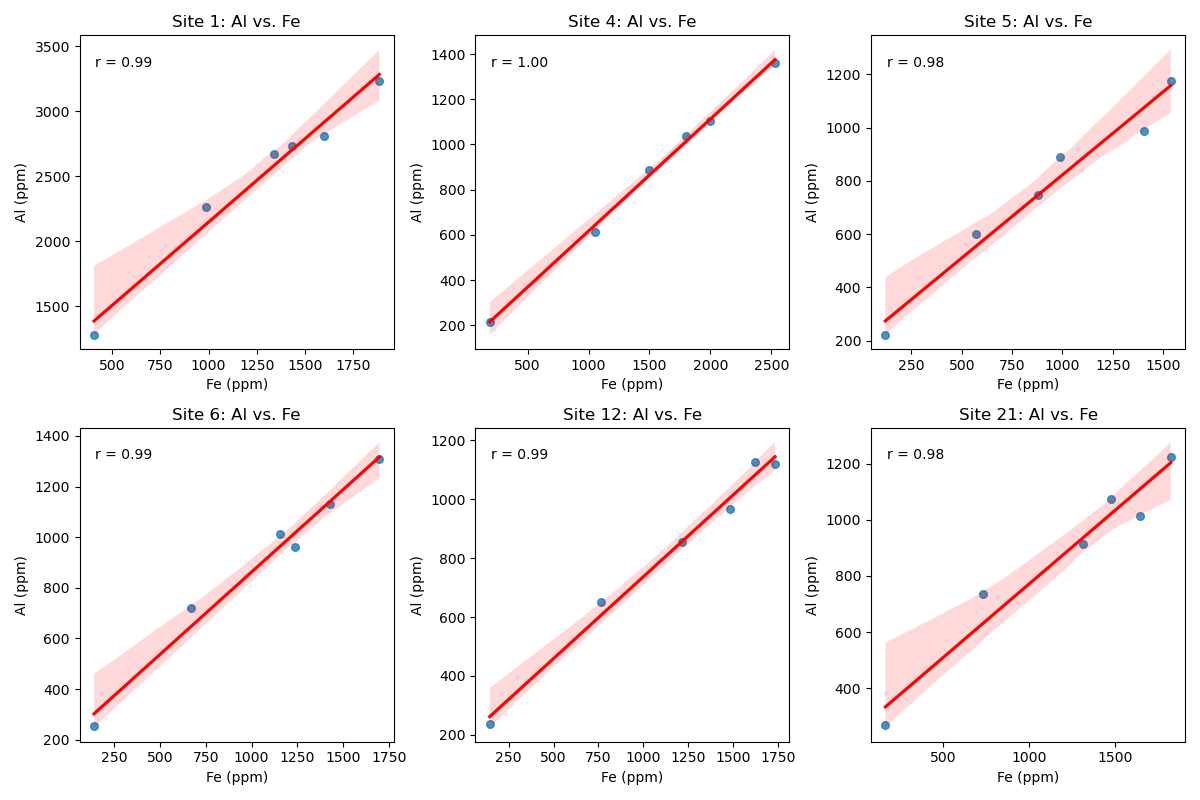


**Figure S25.** Diagrams showing the (Pearson) correlation between the concentrations of Al and Fe (in ppm) in the *simulated gastric-fluid* extracts for each site. Regression lines shown in red, confidence intervals are shaded in red). The correlated increases in the concentrations of Al and Fe from lower left to upper right, seen in each diagram, reflect the compositional evolution of the gastric fluid from the beginning to the end of the experiments (see Figs. 3, S9).


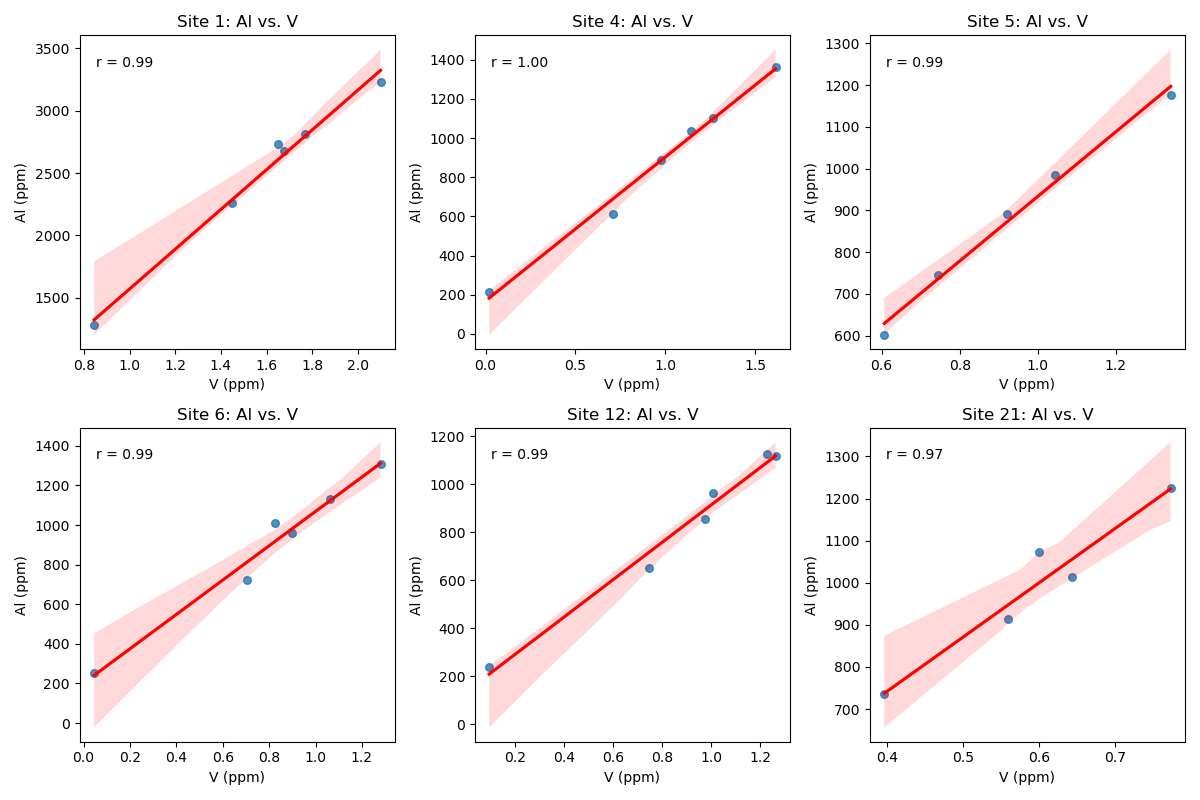


**Figure S26.** Diagrams showing the (Pearson) correlation between the concentrations of Al and V (in ppm) in the *simulated gastric-fluid* extracts for each site. Regression lines shown in red, confidence intervals are shaded in red. The correlated increases in the concentrations of Al and V from lower left to upper right, seen in each diagram, reflect the compositional evolution of the gastric fluid from the beginning to the end of the experiments (see Figs. 3, S11).


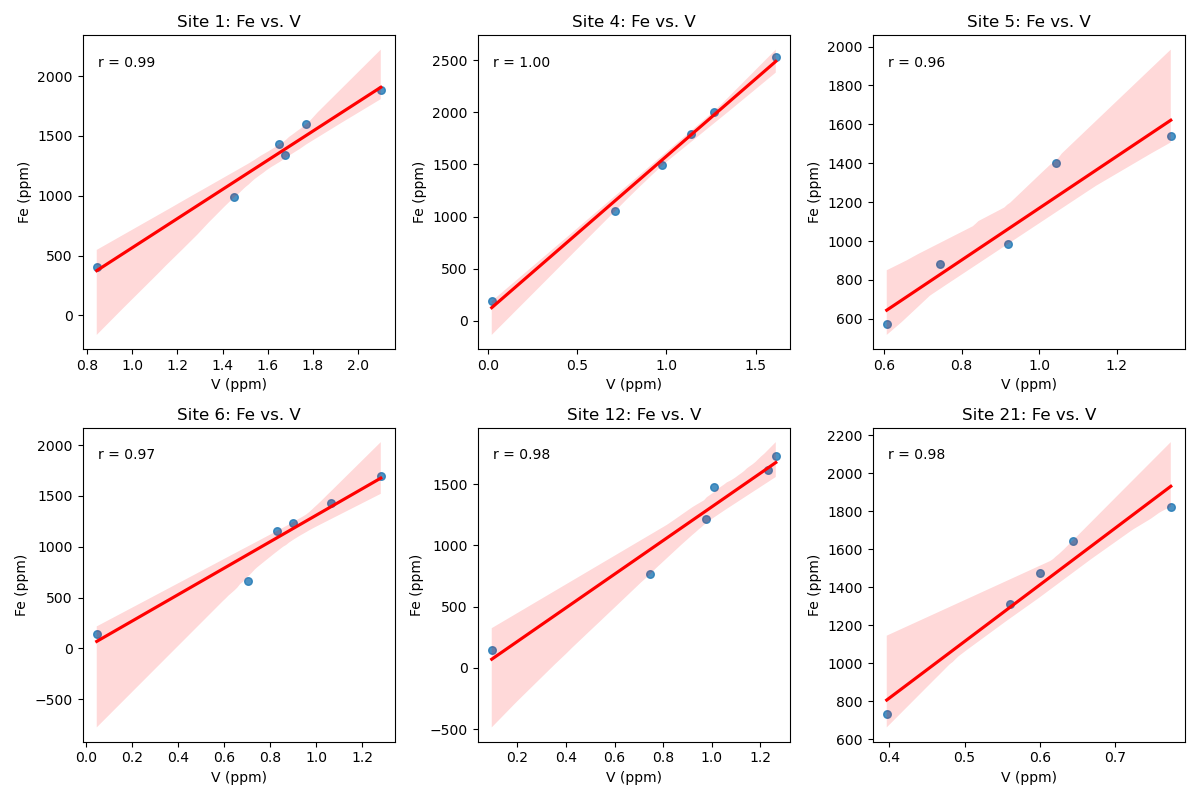


**Figure S27.** Diagrams showing the (Pearson) correlation between the concentrations Fe and V (in ppm) in the *simulated gastric-fluid* extracts for each site. Regression lines shown in red, confidence intervals are shaded in red. The correlated increases in the concentrations of Fe and V from lower left to upper right, seen in each diagram, reflect the compositional evolution of the gastric fluid from the beginning to the end of the experiments (see Figs. S9, S11).


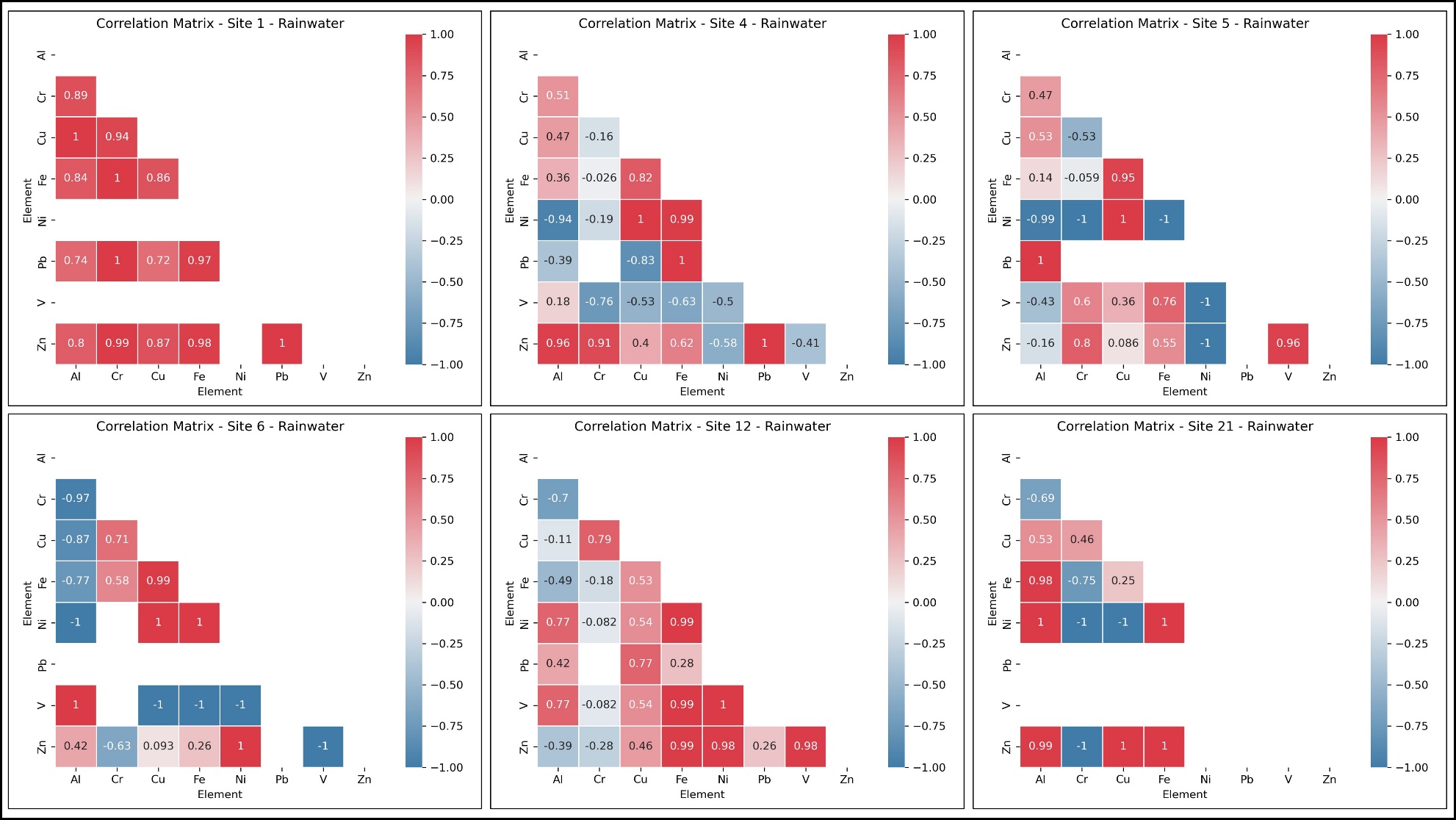


**Figure S28.** Pearson correlation matrices for the concentrations of the studied elements in the *synthetic rainwater* extracts from all six sites. Data points with element concentrations below their respective detection limits were not considered.

**References**

O’Shea MJ, Vann DR, Hwang WT, Gieré R (2020) A mineralogical and chemical investigation of road dust in Philadelphia, PA, USA. Environ Sci Pollut Res 27: 4883-14902. <https://doi.org/10.1007/s11356-019-06746-y>

Berg TM, Edmunds WE, Geyer AR, Glover AD, Hoskins DM, MacLachlan DB, Root SI, Sevon WD, Socolow AA (1980) Geologic map of Pennsylvania (2^nd^ ed.). Pennsylvania Geological Survey. Middletown, Pennsylvania, United States. [https://ngmdb.usgs.gov/Prodesc/proddesc_34341.htm](https://urldefense.com/v3/__https:/ngmdb.usgs.gov/Prodesc/proddesc_34341.htm__;!!IBzWLUs!Xh680TTsxcLhYPjWLxtTWKWACdPbUwXo4xKWrk8bl_tqh-adhN6V6rDw8ofakcwK3hgFVsiNQ7qI8--mNVYIU9Ko$)
